# Probing mixed-state phases on a quantum computer via Renyi correlators and variational decoding

**DOI:** 10.1038/s41467-026-73814-6

**Published:** 2026-06-27

**Authors:** Yuxuan Zhang, Timothy H. Hsieh, Yong Baek Kim, Yijian Zou

**Affiliations:** 1 Department of Physics, University of Toronto, Toronto, ON Canada; 2 Vector Institute, Toronto, ON Canada; 3 Institute of Physics, Ecole Polytechnique Fédérale de Lausanne (EPFL), Lausanne, Switzerland; 4 Department of Physics, Princeton University, Princeton, NJ USA; 5 Perimeter Institute for Theoretical Physics, Waterloo, ON Canada

**Keywords:** Quantum physics, Condensed-matter physics, Information theory and computation, Theory and computation, Computer science

## Abstract

Recent advances have defined nontrivial phases of matter in open quantum systems such as many-body quantum states subject to environmental noise. In this work, we experimentally probe and characterize mixed-state phases on Quantinuum’s H1 quantum computer using two measures: Renyi correlators and the coding performance of a quantum error-correcting code associated with the phase. As a concrete example, we probe the low-energy states of the critical transverse field Ising model under different dephasing noise channels. First, we employ shadow tomography to extract a newly proposed Renyi correlator in two distinct phases obtained by applying two different channels, observing one with faster decay and the other with slower decay. Second, we investigate the decoding fidelity of the associated quantum error-correcting code using a variational quantum circuit. We find that a shallow circuit of depth-3 is sufficient to distinguish the above-mentioned two mixed-state phases, up to *L* = 24. Experimentally, we observe this distinction for system sizes up to *L* = 8 with a depth-1 decoder. Our work is a proof of concept for the quantum simulation and characterization of mixed-state phases.

## Abstract (web summary)

On a trapped-ion quantum computer, the authors combine Rényi correlators with a variational error-correcting decoder to experimentally distinguish mixed-state phases of a noisy critical Ising chain, a frontier of open quantum matter.

## Introduction

Classifying quantum phases of matter has mainly focused on pure states^1^, but realistic devices have noise and lead to open quantum systems and mixed states^2,3^. Information-theoretic definitions for mixed-state phases and their classifications have been developed by recent works^4–9^. It has now been understood that decoherence and measurement can produce nontrivial mixed-state phases, with distinct behaviors in long-range entanglement and critical scaling^10–18^. In the context of topological phases, classification and universal characterization of mixed-state topological order have been partially achieved, highlighting a close relation to quantum error correction^19–26^. Adding symmetries further enriches the classification of mixed-state phases, such as average symmetry-protected topological phases^27–34^ and strong-to-weak spontaneous symmetry breaking^35–41^. When a system undergoes a dissipative process, such as contact with a thermal bath or exposure to quantum noise, the resulting mixed state can exhibit phase transitions that extend beyond traditional pure-state and thermal paradigms. As is the case with gapped and gapless ground state phases, for mixed states, one can also have two varieties depending on whether correlations are short-ranged or critical.

Critical mixed-state phases, which can be obtained by decohering critical ground states^10,12,42^ or measurement and feedforward^11,43^, constitute an important frontier in open quantum systems. One particularly interesting insight of the mixed-state phases comes from quantum error correction codes (QECCs)^44–46^, as the mixed-state phase transitions coincide with decodability transitions in particular QECCs known as CFT codes^47,48^. Mixed state phases and QEC properties are fundamentally connected^6,47^: whether a noisy long-range entangled state remains in the same phase as the pure initial state is related to whether logical information encoded in the long-range entanglement can be decoded from the noisy state. Despite the theoretical formulation, it has been challenging to observe mixed-state phases and their code properties experimentally because they are typically characterized by quantities that are nonlinear in the density matrix, such as the coherent information^47^ or logarithmic negativity^12^.

In this work, we propose two experimentally accessible quantities to detect mixed-state phases on a quantum simulator. The first one is the Renyi correlator, which generalizes conventional correlation functions to integer Renyi index *n* ≥ 2 (for comparison, the two phases could only be distinguished using Renyi negativity at order *n* ≥ 3, which is considerably more demanding experimentally). While Renyi correlators diagnose mixed-state phase transitions associated with a given Rényi index, they may not diagnose intrinsic phase transitions associated with information-theoretic quantities in the replica limit *n* → 1. Thus, to complement Renyi correlators, we utilize the connection with QECCs. We propose a second diagnostic known as the entanglement fidelity^49^. Physically, it quantifies the maximum recoverable information of an encoded quantum state after noise exposure. Given the inherent fragility of quantum states, QEC codes are widely regarded as a cornerstone for achieving fault-tolerant quantum computation and quantum advantage^44–46,50–52^.

**Fig. 1:**
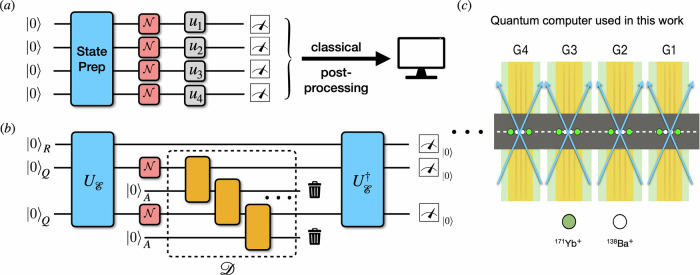
Circuit representation for two experimental setups to distinguish critical mixed state phases. **a** We prepare a quantum critical state described by a variational sequential circuit and numerically find that the state can be prepared at a fidelity ${{\mathcal{F}}} \sim 0.99$ with 5 SU(4) gates at *L* = 6. We then apply the noise channel (local dephasing on each site) and compute the Renyi correlator with shadow tomography, see SM for more details. **b** We regard the CFT as a QECC with its ground state and first excited state as the codewords and diagnose mixed-state phases using the QEC property. The decoder is implemented as a variational circuit with ancilla, as shown in the diagram. The circuit is optimized to maximize the entanglement fidelity *F*_*e*_. **c** The architecture of the quantum computer (Quantinuum H1), which was first introduced in ref. ^74^. The Quantinuum H1 processor features 20 qubits arranged in 1D, and with possibility of performing physical ion moves, it enables all-to-all connectivity, supporting up to five simultaneous two-qubit operations in parallel. Only nearest neighbor gates are used for the purpose of this work.

We demonstrate our proposal on a trapped-ion quantum processor using 4–8 spins. The two complementary experimental setups—measuring the Rényi correlator and the entanglement fidelity of the variational decoder—are illustrated in Fig. 1. Specifically, we prepare the ground state of the critical transverse field Ising model using a variational circuit and subject them to dephasing noise. We then use randomized measurement techniques^53,54^ to estimate Renyi correlators. We observe different behaviors of Renyi correlators depending on the direction of the dephasing, where *X* dephasing enhances the the Renyi correlator to slower decay and *Z* dephasing reduces the Renyi correlator to faster decay, in agreement with CFT predictions. In order to measure the entanglement fidelity, we construct a decoder for the CFT code using a variational quantum channel. Using Stinespring’s dilation theorem, the decoding channel is realized by a variational unitary circuit acting on both physical and ancilla qubits, where the unitary gates are determined through a classical optimization. Our simulation indicates that the two mixed-state phases with up to *L* = 24 spins can be distinguished using shallow, depth-3 decoding circuits. We find that the entanglement fidelity decreases monotonically with *L* For the *X* dephasing, whereas the *Z* dephasing shows a non-monotonic behavior, that *F*_*e*_ decreases with *L* at small fixed *pL*^1/2^ and increases with *L* at large fixed *pL*^1/2^. On the current-stage quantum simulator, we observe this distinction up to *L* = 8 spins using depth-1 decoders despite hardware constraints. Both the Rényi correlator and entanglement fidelity confirm the experimental observation of two distinct mixed-state critical phases at Renyi index *n* = 2 and the replica limit. We note that *Z* dephasing corresponds to different kinds of perturbations in the renormalization group (RG) flow depending on the Renyi index: this perturbation is marginal for Renyi index 2 and irrelevant for the replica limit^47^.

## Results

### Setup and Renyi correlators

We consider the ground state $| \psi \rangle$ of the critical transverse-field Ising model

$$H=-{\sum }_{i=1}^{L}{X}_{i}{X}_{i+1}-{\sum }_{i=1}^{L}{Z}_{i}$$

subject to noise ${{\mathcal{N}}}={\otimes }_{i}{{{\mathcal{N}}}}^{[i]}$, where ${{{\mathcal{N}}}}^{[i]}$ the dephasing channel in *X* or *Z* directions on site *i*. In ref. ^12^, it was shown that the different negativity scaling between two mixed states indicates a mixed-state phase transition. In this work, we show that the distinction also manifests in the Rényi correlators for *n* ≥ 2 (which permits more efficient experimental detection). Given a mixed state *ρ*, we define the *n* − th Renyi observables through

1 $${\langle O\rangle }^{(n)}=\frac{{{\rm{tr}}}({\rho }^{n}O)}{{{\rm{tr}}}({\rho }^{n})},$$

where *O* is an operator and *n* is a positive integer (not to be confused with a similar quantity studied in refs. ^10,18,39,41,55^ to detect spontaneous strong to weak symmetry breaking). The Renyi observable reduces to the usual expectation value ${\langle O\rangle }^{(1)}={{\rm{tr}}}(\rho O)$ for *n* = 1. At *n* = 2, the Renyi correlator is the expectation value of *O*⊗*I* on the vectorized state $| \rho \rangle \rangle$ in the doubled Hilbert space. This doubled Hilbert space picture has been used to characterize mixed-state phases theoretically^34,,^. We will consider the Renyi two-point correlator, ${C}_{{O}_{1}{O}_{2}}^{(n)}(l):={\langle {O}_{1}^{[0]}{O}_{2}^{[l]}\rangle }^{(n)}$, where *O*_1_ and *O*_2_ are local operators on site 0 and site *l*, respectively.

**Fig. 2:**
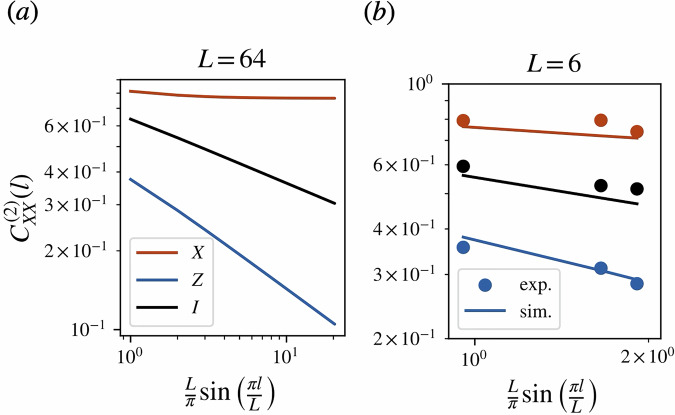
Renyi correlators. **a** ${C}_{XX}^{(2)}$ for the Ising ground state under different dephasings, with total system size *L* = 64 and *p* = 0.3. For *X* dephasing the correlator saturates to a constant. For *Z* dephasing the correlator has a faster power-law decay, with ${\eta }_{2}^{Z}=0.35(1)$ compared to ${\eta }_{2}^{I}=1/4$ with no noise. The different behaviors of the two dephasings are robust across all *p* (see SM). **b** Experimental results on the Quantinuum H1 trapped-ion processor with *L* = 6 and *l* = 1, 2, 3. The line represents exact simulation result, and the dots represent experimental data. We use 40k samples per data point, and a random unitary was drawn for each circuit run. A jackknife estimation (similar to^54^) reports that the error bars are less than 1 percent, showing that the deviation is mostly due to device noise, including logical gate error and SPAM error, which we further justify in the SM. The experimental estimation for the slope is 0.31(7) for *Z*, 0.07(8) for *X*, and 0.21(8) for the identity channel, due to finite-size corrections and noise.

In the thermodynamic limit, the Renyi *XX* correlator at long distances takes the form of,

2 $${C}_{XX}^{(n)}(l)={A}^{(n)}+{B}^{(n)}{\left(\frac{L}{\pi }\sin \left(\frac{\pi l}{L}\right)\right)}^{-{\eta }_{n}},$$

where *A*^(1)^ = 0 and *η*_1_ = 1/4 is the critical exponent of the Ising model for both dephasing channels. The correlator shows distinctive properties for the *X* and *Z* channels at *n* ≥ 2. For the *X*-dephased state, the correlator becomes long-range, i.e., *A*^(*n*)^ > 0. For the *Z*-dephased state *A*^(*n*)^ = 0 and *η*_*n*_ decreases continuously for *n* ≥ 2. This is verified through the numerical simulation on *L* = 64 spins shown in Fig. 2a. The Ising model is known to have small finite-size effects, and the CFT prediction is emerging already at small sizes such as *L* = 6, as shown in Fig. 2b. For example, one can numerically extract the scaling dimensions of the spin operator *σ* and energy operator *ε* from the energy spectrum: at *L* = 8 sites, the numerical values are Δ_*σ*_ = 0.130 and Δ_*ε*_ = 1.03, agreeing within 4% relative error of the CFT predictions (0.125 and 1, respectively), and this finite-size correction is further suppressed as 1/*L*^2^ with increasing size.

The result can be explained through the mapping from decoherence channels to conformal defects^10,12,56,57^. The exponents *η*_2_ give exactly twice the scaling dimensions of defect operators in the replicated CFT. For *X* dephasing, it induces a relevant spin-spin coupling on the defect, making the defect long-range ordered such that *A*^(2)^ > 0. The *Z* dephasing is a marginal perturbation, such that *η*_2_ continuously changes with dephasing rate *p*. Interestingly, our numerical fit suggests that *η*_2_ ≈ 5/4 for the subleading exponent in ${C}_{XX}^{(2)}$ under *X* dephasing, whereas the naive expectation that the defect is factorized into a direct sum of polarized boundaries predicts that *η*_2_ = 4. We also computed other Renyi correlators, which show interesting new exponents, see SM for details.

### Shadow tomography

We use the randomized measurement method^54^ known as shadow tomography to reconstruct Renyi correlators from experimental data. One key advantage of shadow tomography is that, after measuring and recording the data, one can extract various non-commuting quantum information quantities, such as the Rényi correlator, from the same data set. We briefly review the method here: For each copy of the state, we first perform a randomized measurement by applying single-qubit unitaries *u*_1_ ⊗ *u*_2_ ⊗ ⋯ ⊗ *u*_*L*_, each sampled independently from an ensemble that forms a unitary 3-design^54,58^ and record the classical measurement outcomes ***k*** = {*k*_1_, …, *k*_*L*_}. The experiment is repeated *M* times, and we denote each set of measurement outcomes as ***k***^(*r*)^, from which we can construct an unbiased estimator of the density matrix.

3 $${\widehat{\rho }}^{(r)}={\bigotimes }_{i=1}^{L}{\widehat{\rho }}_{i}^{(r)}={\bigotimes }_{i=1}^{L}\left(3{u}_{i}^{{\dagger} }\left| {{{\boldsymbol{k}}}}_{i}^{(r)}\right\rangle \left\langle {{{\boldsymbol{k}}}}_{i}^{(r)}\right| {u}_{i}-{{\mathcal{I}}}\right),$$

whose expectation gives raise to *ρ*^53^. For *n* = 2 Renyi observables, we find that

4 $$\langle {{\rm{tr}}}({\rho }^{2}O)\rangle=\frac{1}{2!}\left({\scriptstyle{M}\atop{2}}\right)^{-1}{{\rm{tr}}}\left\{{\sum }_{r}{[{\widehat{\rho }}^{(r)}]}^{2}O-{\sum }_{r}[{({\widehat{\rho }}^{(r)})}^{2}]\left)\right. O\right\},$$

To increase the quality of data further, we use the system’s translational symmetry. Namely, we permute the qubits and construct independent estimations of ${C}_{XX}^{(2)}(l)$ and report their mean value as the estimation of ${C}_{XX}^{(2)}(l)$.

The experimental procedure is as follows:

1. Prepare the critical Ising ground state with a variational quantum circuit.
2. Apply the dephasing quantum channel: a Pauli operator is applied to each site with probability *p*.
3. Apply a random single qubit rotation *u*_*i*_, independently sampled from a 3-design (as we only compute quantities up to the third moment of the density matrix), to each physical qubit.
4. Measure in the computational basis and record the measurement result.
5. Repeat 1–4 until the desired data set size is reached.
6. Classical post-process to get an estimation of the quantum information quantities.

We implement this experimental procedure for both the *X* and *Z* dephasing with a strength of *p* = 0.3 on Quantinuum’s H1 trapped-ion quantum computer (with 40,000 circuit runs each) and report the measured Renyi correlators in Fig. 2; in the Supplementary Material we also compute the Renyi negativity and find agreement with the theoretical expectations. Our results indicate that the experimental setup recovers Rényi correlators within an inaccuracy of ~10% compared to exact diagonalization (ED) (see also SM for a shadow estimation of Renyi negativity). The error mainly comes from the device noise, including two-qubit gates and measurement error.

We observe clear distinctions between the state under different channels, i.e., X dephasing, Z dephasing, and identity channel. In terms of the amplitude of ${C}_{XX}^{(2)}(l)$, we observe a significant increase under *X* dephasing and a significant decrease under the *Z* dephasing w.r.t. the identity channel. Furthermore, we can approximately extract the slope of the decay of ${C}_{XX}^{(2)}(l)$ on the logarithmic scale using the data with *l* = 1, 2, 3 on the *L* = 6 chain. We fit the slope 0.31(7) for *Z* dephasing, 0.07(8) for *X* dephasing, and 0.21(8) for the identity channel, respectively. The result is consistent with the field-theoretic prediction that slope for *Z* dephasing is greater than 0.25 (the predicted slope for the identity channel) and the slope for *X* dephasing is 0 as *L* → *∞*. These findings suggest that even at this relatively small system size, signatures of two distinctive mixed-state phases induced by noise are observed on a quantum computer.

### Decoding a CFT code

Now we turn to the second diagnostic, which utilizes the decodability of the CFT code to observe the aforementioned two mixed-state critical phases. Specifically, one could encode *D* logical states using the low-energy subspace spanned by the *D* lowest-energy eigenstates, $| {\phi }_{\alpha }\rangle$ of the critical Hamiltonian *H*. The CFT code may have a finite decoding threshold *p*_*c*_ ≠ 0 in the thermodynamic limit, albeit at finite sizes the code becomes approximate^47,48^. In particular, the Ising CFT code can correct uniform *Z* dephasing error for *p* < 1, but has a zero decoding threshold for the *X* dephasing error.

Formally, let *R* be a *D*-dimensional reference qudit and the code subspace spanned by $\{{| {\phi }_{\alpha }\rangle }_{Q},0\le \alpha \le D-1\}$ and the maximal entangled state $| {\phi }_{RQ}\rangle=\frac{1}{\sqrt{D}}{\sum }_{\alpha=0}^{D-1}{| \alpha \rangle }_{R}{| {\phi }_{\alpha }\rangle }_{Q}$, where *Q* is the set of physical qubits. Throughout the rest of the paper, we set *D* = 2 for consistency. Let ${{{\mathcal{N}}}}_{Q}={\otimes }_{j\in Q}{{{\mathcal{N}}}}^{[j]}$ be the noise channel acting on *Q* and we can define the noisy state

5 $${\rho }_{RQ}={{{\mathcal{N}}}}_{Q}(| {\phi }_{RQ}\rangle \langle {\phi }_{RQ}| )$$

We will use *entanglement fidelity*, *F*_*e*_, to quantify the decodability of the CFT code, which is defined as:

6 $${F}_{e}={\max }_{{{\mathcal{D}}}}\langle {\phi }_{RQ}| {{{\mathcal{D}}}}_{Q}({\rho }_{RQ})| {\phi }_{RQ}\rangle .$$

where ${{{\mathcal{D}}}}_{Q}$ is a decoding channel acting on *Q*. Since the explicit construction of decoders is unknown, we propose a greedy algorithm to compute *F*_*e*_ for the CFT code at small system sizes and compare it with the bounds defined below. Specifically, we introduce ancilla qubits, *A*, and dilate the channel ${{{\mathcal{D}}}}_{Q}$ into an isometry *W*: *Q* → *QA* such that ${{{\mathcal{D}}}}_{Q}(\rho )={{{\rm{tr}}}}_{A}W\rho {W}^{{\dagger} }$. The entanglement fidelity can then be written as

7 $${F}_{e}(W,{W}^{{\dagger} })=\langle {\phi }_{RQ}| {{{\rm{tr}}}}_{A}\{W{\rho }_{RQ}{W}^{{\dagger} }\}| {\phi }_{RQ}\rangle .$$

The optimization is as follows: at each step, we keep *W*^†^ fixed and compute the maximum of *F*_*e*_(*W*, *W*^†^) over *W*, and then compute the maximum over *W*^†^ by fixing *W*. The step is repeated until *F*_*e*_ converges. We typically find that *F*_*e*_ converges in less than 5 iterations, see SM for details.

Optimizing *F*_*e*_ for large systems soon becomes formidable, but one may bound *F*_*e*_ through various quantities that are easier to compute. One quantity is the channel distance, defined as

8 $${d}_{\rho }=\sqrt{1-f({\rho }_{RE},{\rho }_{R}\otimes {\rho }_{E})},$$

where ${\rho }_{RE}={{{\rm{tr}}}}_{Q}{\rho }_{RQE}$. Here *ρ*_*RQE*_ is a purification of the state *ρ*_*RQ*_ with environment *E* coming from the dilation of the noise channel ${{\mathcal{N}}}$, and *f* is the Uhlmann fidelity. The channel distance is independent of the choice of purification and provides a lower and upper bound on *F*_*e*_ in the following way^59^:

9 $$\frac{1}{2}{d}_{\rho }\le \sqrt{1-\sqrt{{F}_{e}}}\le {d}_{\rho }$$

**Fig. 3:**
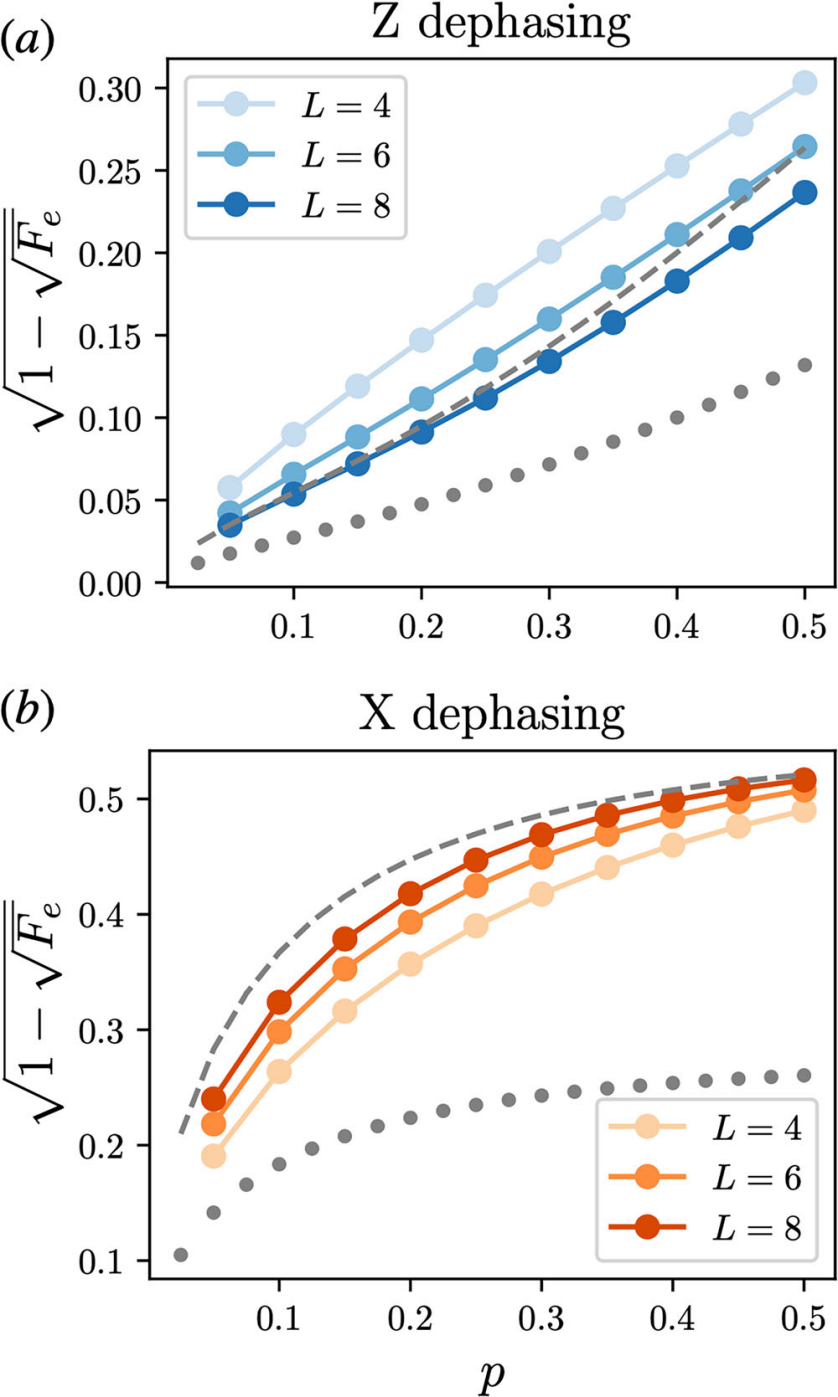
Optimal entanglement fidelity from numerical simulations. **a** Optimal entanglement fidelity *F*_*e*_ of the Ising CFT code under *Z* dephasing versus channel distance *d*_*ρ*_ in Eq. (8). **b** Same quantity under *X* dephasing. In both panels, the dotted and dashed grey lines represent *d*_*ρ*_ and *d*_*ρ*_/2 at *L* = 8, which are upper and lower bounds for $\sqrt{1-\sqrt{{F}_{e}}}$, respectively. The upper bound *d*_*ρ*_ is saturated for *Z* and *X* dephasings at small and large *p*, respectively. The numerical calculation is performed with exact diagonalization.

For *L* = 4, 6, 8, we compute optimal entanglement fidelity *F*_*e*_ numerically and compare it with the bound in Fig. 3. The optimized entanglement fidelity *F*_*e*_ is consistent with the bound Eq. (9) and saturates the upper bound for weak *Z* dephasing. Additionally, for the *Z* dephasing, *F*_*e*_ increases with *L* for fixed *p*, indicating a finite decoding threshold, as logical information is better preserved by adding more physical qubits. For the *X* dephasing, *F*_*e*_ decreases with *L* for fixed *p*, indicating zero decoding threshold. Nevertheless, we note that the CFT code can still correct subextensive number of *X* errors if *p* < *O*(*L*^−3/4^). As we will see, this large gap between the number of correctable errors in *X* and *Z* dephasing is useful for the experimental detection of the two mixed-state phases using variational decoding.

### Variational decoding

In order to implement the decoder on a quantum computer, we resort to a variational approach where the channel is dilated into a variational unitary circuit with ancillas. By the HJW theorem, any mixed state can be purified with twice the number of qubits, so the mapping between two such states can be implemented with a unitary operation using one ancilla qubit per system qubit. Concretely, we introduce an ancilla qubit between each nearest-neighbor pair of system qubits and construct a ladder-shaped variational quantum circuit. This circuit structure is first proposed as a quantum realization of the matrix product state^60–67^, and is illustrated in Fig. 1b. The ladder structure is more expressive than brick-wall structure when comparing circuits with the same gate count, as noted in recent works^68,69^. For Quantinuum H1 processor’s architecture, the main error source comes from entangling gates; this reduced gate count makes the sequential circuit more favorable (see SM for more details).

**Fig. 4:**
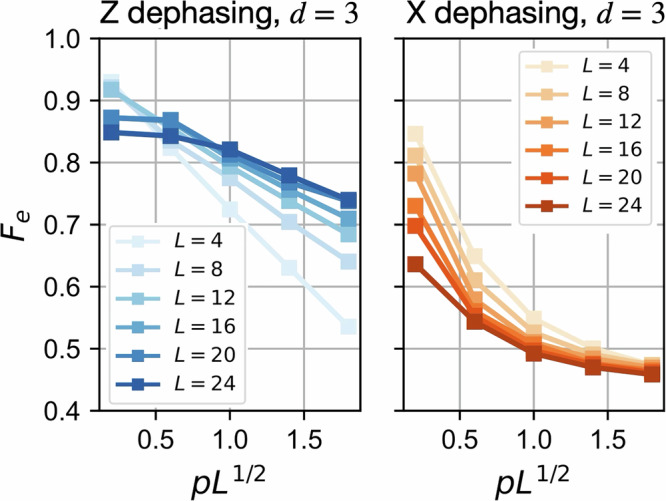
Entanglement fidelity from numerical simulation of variational decoding. For a range of system sizes *L* = 4, 8,... 24, the noise rate is chosen to be *p* ∝ *L*^−1/2^. fixing constant decoding depth *d* = 3, we observe that the shallow variational decoder is already correcting the non-extensive Z error, resulting in the increase of *F*_*e*_ after the decoding step, whereas the X error remains uncorrectable.

For each noise rate *p* and dephasing direction, we classically optimize the gates in the decoding circuit to maximize the entanglement fidelity *F*_*e*_. For small system sizes, we observe that the optimal decoding depth *d* increases quickly with *L*, which hinders experimental observation on near-term devices. Nevertheless, we find that a sub-optimal decoder at a shallow depth is already sufficient to distinguish the two mixed-state phases when *p* ∝ *L*^−1/2^: this noise rate remains uncorrectable for the *X* dephasing and correctable for *Z*. We use tensor network to compute the variational decoding fidelity *F*_*e*_ (See SM for numerical details) up to circuit depth *d* = 3 and *L* = 24 spins, as shown in Fig. 4. For fixed *pL*^1/2^, *F*_*e*_ is monotonically decreasing with *L* for the *X* dephasing but increase with *L* for the *Z* dephasing when *pL*^1/2^ ≿ 1. The latter non-monotonic behavior of *F*_*e*_ comes from the competition between limited decoding depth and the increase of optimal decoding fidelity with *L*. This reflects the difference of decodablility in the thermodynamic limit, in agreement with the CFT prediction.

**Fig. 5:**
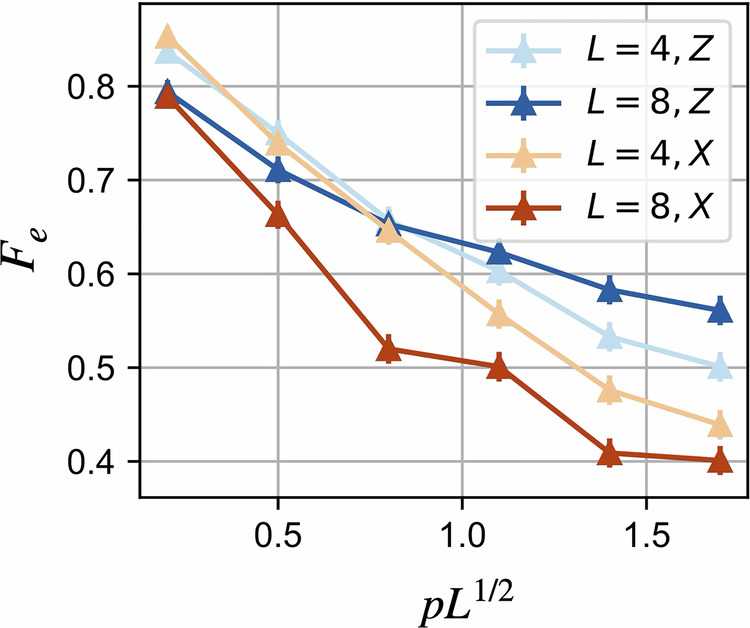
Experimental results on variational decoding. Experimental results on entanglement fidelity *F*_*e*_ of the Ising CFT code with *L* = 4 and *L* = 8 with a single-layer decoder (1000 shots), with *p* ∝ *L*^−1/2^. Error bars represent one standard deviation due to finite sampling. For *Z* dephasing at large *p*, *F*_*e*_ increases with *L*, which persists even with device noise. Whereas for *X* dephasing, *F*_*e*_ strictly decreases with *L* even if the error rate *p* ∝ *L*^−1/2^. The decoding circuits come from classical optimization for each noise rate and are implemented experimentally.

In the experiment, we run the variational circuit and obtain the entanglement fidelity *F*_*e*_ with small systems *L* = 4, 8 and decoding circuit depth *d* = 1. *F*_*e*_ is measured through the frequency of obtaining all 0 output string in the final measurement. The result is shown in Fig. 5. We see that the entanglement fidelity *F*_*e*_ for the *Z* dephasing shows the expected non-montonic behavior as one increases *L* while keeping *pL*^1/2^ fixed. The *X* dephasing gives monotonically decreasing *F*_*e*_. Thus, the experiment successfully distinguish the decodable versus nondecodable phases up to *L* = 8, even in the presence of additional physical noise. For *L* = 8 we estimate the physical noise introduces a ~9% deviation; see SM for more details. We also estimate in SM that in order for the distinction to be observed between, e.g., *L* = 8 and *L* = 24, we need the fidelity of each native two-qubit gate to be 10^−4^, which can be achievable in the near future.

Compared with Renyi observables, which require exp(O(L)) samples for shadow tomography (measuring the Renyi correlator with a SWAP test also requires exponential sampling, since the purity is exponentially small in *L*), the sampling complexity for variational decoding is *O*(1) when the decoder is known. When the decoder is not known, one could implement a transfer learning strategy to ensure a non-exponentially-vanishing signal during initialization (see SM for more details). We emphasize that this transfer-learning approach relies on the decoder being *local* and on the optimal-decoder parameters evolving smoothly with system size *L*, so that an optimum at *L* − 1 provides a near-optimal seed at *L*. Furthermore, as we demonstrate both numerically and experimentally, a shallow decoding depth is enough to distinguish the two decoding phases, making the variational decoding approach more favorable.

## Discussion

In this work, we have demonstrated that mixed-state phases in critical quantum systems can be observed experimentally using two complementary diagnostic tools: Renyi correlators and variational decoding. We employed shadow tomography to measure second-order Rényi two-point correlators that revealed distinct power-law behaviors under different dephasing channels. The other approach, based on variational decoding, optimizes the entanglement fidelity of the QECC based on the low-energy subspace of the same quantum system, successfully distinguishing the two error-correcting phases induced by *X* and *Z* dephasing channels even at shallow circuit depths. Similar results are expected to hold for any lattice model that realizes the Ising CFT: the noise is not correctable if the Kraus operators has nonzero overlap with the spin operator and correctable if the Kraus operators respect the strong *Z*_2_ symmetry.

While Rényi correlators require no ancilla qubits to measure, they detect phase transitions specific to integer Rényi index. On the other hand, entanglement fidelity probes the information-theoretic phase transition^47^, capturing transitions that are otherwise challenging to observe. This distinction makes these two measures complementary experimental tools for characterizing mixed-state phases in open quantum systems.

Looking ahead, our work opens several promising directions for future research. First, from a field-theoretic standpoint, it is intriguing to explore the universal properties of the decoherence-induced defect and to develop an analytical understanding of the associated Rényi correlators. A second open problem is the analytical study of optimal decoders for CFT-based quantum codes considered in this work. Third, techniques such as shadow tomography and variational decoding could be leveraged to experimentally observe other mixed-state phases, including symmetry-protected topological (SPT) phases (see SM for an example) and topologically ordered states, where information recoverability serves as the key characterization of mixed-state phases. Lastly, by combining variational decoding with machine learning, it may be possible to discover error-correcting protocols capable of mitigating physical noise–even in cases where the noise model is unknown or ill-defined.

## Methods

The experiments use a variational sequential SU(4) circuit on the Quantinuum H1 trapped-ion processor to prepare the critical Ising ground state (${{\mathcal{F}}} \sim 0.99$ at *L* = 6 with 5 gates), followed by a single-qubit Pauli-*X* or Pauli-*Z* dephasing channel applied with probability *p* per site. Renyi correlators are extracted via classical shadow tomography with single-qubit 3-design unitaries and jackknife error bars, and entanglement fidelity *F*_*e*_ is read out from the all-zero outcome frequency at the output of the decoder. Below we focus on the technical methodology that supports these measurements: the optimal-decoder benchmark used at small system sizes, the MPS construction of the CFT codeword, and the variational and transfer-learning strategies used to optimize the decoding circuit at moderate *L*. Further implementation details, the noise model, and supporting numerics are given in the Supplementary Material.

### Optimal decoder through repeated SVD

At small system sizes, we compute the optimal entanglement fidelity directly. Consider the state $| {\phi }_{RQ}\rangle$ under decoherence ${\rho }_{RQ}={{{\mathcal{N}}}}_{Q}(| {\phi }_{RQ}\rangle \langle {\phi }_{RQ}| )$. The entanglement fidelity is

10 $${F}_{e}={\max }_{W}\langle {\phi }_{RQ}| {{{\rm{tr}}}}_{A}(W{\rho }_{RQ}{W}^{{\dagger} })| {\phi }_{RQ}\rangle,$$

where *W*: *Q* → *QA* is an isometry with *W*^†^*W* = *I*. We optimize *W* by rewriting

11 $${F}_{e}={\max }_{W}{{\rm{tr}}}\,{E}_{W}^{T}W,\,{E}_{W}^{T}={{{\rm{tr}}}}_{R}({\rho }_{RQ}\,{W}^{{\dagger} }| {\phi }_{RQ}\rangle \langle {\phi }_{RQ}| ).$$

Viewing *E*_*W*_ as a matrix from *Q* to *QA* and performing an SVD *E*_*W*_ = *USV*^†^ with *U*^†^*U* = *V*^†^*V* = *I*, the update rule is *W* ← U^*^V^*T*^ and the updated maximum is ${F}_{e}\leftarrow {{\rm{tr}}}\,S$. We initialize *W* as a random isometry and iterate; in practice, *F*_*e*_ converges to five digits within five iterations. The ancilla dimension *d*_*A*_ = *d*_*Q*_ suffices to achieve the optimum. The cost of one iteration is dominated by computing *E*_*W*_ (scaling as $O({d}_{R}^{2}{d}_{Q}^{2}{d}_{A})$) and the SVD step ($O({d}_{Q}^{3}{d}_{A})$); for *L* = 8 system qubits and one reference qubit on a laptop with an Intel i7-10875H processor, each iteration takes roughly 8 seconds.

### MPS construction for the CFT code

To study the CFT code at large *L* we represent the codeword $| {\phi }_{RQ}\rangle=\frac{1}{\sqrt{2}}({| 0\rangle }_{R}{| {\phi }_{0}\rangle }_{Q}+{| 1\rangle }_{R}{| {\phi }_{1}\rangle }_{Q})$ as a matrix product state. Using DMRG we obtain MPS representations of the ground state $| {\phi }_{0}\rangle$ and first excited state $| {\phi }_{1}\rangle$ of length *L*, with tensors *A*^[*j*]^(*s*_*j*_) and *B*^[*j*]^(*s*_*j*_) of bond dimensions (1, *D*_1_, …, *D*_*L*−1_, 1) and (1, *E*_1_, …, *E*_*L*−1_, 1), respectively. We then construct an (*L* + 1)-site MPS {*C*^[*j*]^} by introducing a reference site (site 1) carrying the logical index, identity-coupling it to a fresh bond, and embedding the two ground-state branches into block-diagonal tensors on the physical sites 2, …, *L* + 1. Concretely, on the first physical site we set *C*^[2]^(*a*_0_, *s*_0_, *b*_0_) equal to $\frac{1}{\sqrt{2}}{A}^{[0]}({s}_{0})$ for *a*_0_ = 0 and $\frac{1}{\sqrt{2}}{B}^{[0]}({s}_{0})$ for *a*_0_ = 1, with the outgoing bond split between the two branches; on intermediate sites *C*^[*j*+2]^(*s*_*j*_) is the block-diagonal diag(*A*^[*j*]^(*s*_*j*_), *B*^[*j*]^(*s*_*j*_)); on the final site the two block tensors are stacked vertically. Because $| {\phi }_{0}\rangle$ and $| {\phi }_{1}\rangle$ are normalized and mutually orthogonal, the prefactor $1/\sqrt{2}$ at site 1 yields a normalized superposition.

### Gradient-based variational optimization

For the variational decoder, each two-qubit gate in the ladder circuit is parameterized as a general SU(4) gate^70^ and the parameters are optimized using PyTorch’s automatic-differentiation framework with the ADAM optimizer^71^. The entanglement fidelity *F*_*e*_ is evaluated by reversing the encoding circuit and reading the probability of the all-zero output string. To mitigate the barren-plateau issue at deep depths, we use a warm-start strategy (see Box 1): starting from a shallow decoder we iteratively grow the depth, using the previously optimized parameters as initialization for the new layers^72,73^; after reaching the target depth we sweep back to obtain decoders at intermediate depths in an over-parameterized regime. We find this consistently outperforms random initialization.

### Transfer learning across system size

For experimental implementation at moderate *L* we further mitigate the barren plateau by transferring optimized parameters between system sizes (see Box 2). Because the decoder is local and correlations evolve smoothly with *L*, the optimum at *L* − 1 is a near-optimal seed at *L*, yielding a controlled finite-size flow. Let *θ*^(*L*)^ denote the variational parameters and ${F}_{e}^{(L)}(\theta )$ the objective. We initialize the larger system from the optimized smaller system via

12 $${\theta }^{(L,0)}={{{\mathcal{T}}}}_{L-1\to L}({\theta }^{(L-1,\star )}),\,{\theta }^{(L,\star )}=\arg {\min }_{\theta }{F}_{e}^{(L)}(\theta ),$$

copying shared parameters and initializing newly introduced gates to identity. We find that the transferred initial *F*_*e*_ is consistently high over *L* = 11–20, in stark contrast to random initialization (Supplementary Material).

Box 2 Algorithm 2 – Transfer learning across system size 1. Train the base model at $L={L}_{\min }$ to obtain ${\theta }^{({L}_{\min },\star )}$.

2. For $$L={L}_{\min }+1,\ldots,{L}_{\max }$$:

(a) Compute *θ*^(*L*, 0)^ ← TRANSFERINIT(*θ*^(*L*−1, ⋆)^, *L*, *d*).

(b) Minimize ${F}_{e}^{(L)}(\theta )$ initialized at *θ*^(*L*, 0)^.

(c) Store the optimized parameters as *θ*^(*L*, ⋆)^.

## Supplementary information

Supplementary Information

Transparent Peer Review file

## Data Availability

The data underlying all main-text and Supplementary Figs. are publicly available at https://github.com/yuxuan-zhang/CFT_channel.

## Code availability

The code used in this study is publicly available at https://github.com/yuxuan-zhang/CFT_channel.
