# Supplementary material for: Probing mixed-state phases on a quantum computer via Renyi correlators and variational decoding
Source: Nat Commun. 2026 Jun 27;17:8047. doi: 10.1038/s41467-026-73814-6 (PMC13454128; doi:10.1038/s41467-026-73814-6)
Supplement: Supplementary file 1 [file 41467_2026_73814_MOESM1_ESM.pdf]

Supplementary Information for "Probing mixed-state phases on a quantum computer via Renyi correlators and variational decoding"

Yuxuan Zhang,[1, 2, 3, 4] Timothy H. Hsieh,[5] Yong Baek Kim,[1] and Yijian Zou[5]

[1] *Department of Physics, University of Toronto, Toronto, Ontario, Canada*
[2] *Vector Institute, Toronto, Ontario, Canada*
[3] *Institute of Physics, Ecole Polytechnique Fédérale de Lausanne (EPFL), Lausanne, Switzerland*
[4] *Department of Physics, Princeton University, Princeton, New Jersey, USA*
[5] *Perimeter Institute for Theoretical Physics, Waterloo, Ontario, Canada*

(Dated: May 13, 2026)

Contents

Different exponents in Renyi correlators 1
Field-theoretic intepretation 1
X dephasing 2
Z dephasing 4

Optimal fidelity of decoding at small sizes 4

Additional variational decoding results 6
Depth-1 decoder at additional system sizes 7
Transfer learning performance 7

Variational decoding for symmetry-protected topological phases 7
Exact decoder at zero transverse field 7
Variational decoding at nonzero transverse field 9

Experimental details 10
Quantinuum H1 Processor 10
Implementation of quantum randomness 10

Classical shadow estimation of Renyi negativity 11

References 12

Different exponents in Renyi correlators

In this section we present numerical simulations of Renyi correlators of the critical TFIM under X or Z noise with $L = 64$ spins and fit for their critical exponents.

Field-theoretic intepretation

The mixed state ρ is obtained through a pure state under local decoherence $\rho = \mathcal{N}(|\psi\rangle\langle\psi|)$, where $\mathcal{N}$ is a product of local channels, $\mathcal{N} = \otimes_j \mathcal{N}^{[j]}$. As one varies the parameters of the local channels, one observes distinct behaviors between Renyi correlators with $n = 1$ and $n \geq 2$. The $n = 1$ correlator can be related to two-point function of the original pure state through $C^{(1)}_{O_1 O_2}(l) = \langle O_1^{[0]} O_2^{[j]} \rangle^{(1)} = \mathrm{tr}\Big(\rho O_1^{[0]} O_2^{[j]}\Big) = \mathrm{tr}\Big(\mathcal{N}(|\psi\rangle\langle\psi|) O_1^{[0]} O_2^{[j]}\Big) = \mathrm{tr}\Big(\mathcal{N}^*(O_1^{[0]} O_2^{[j]}) |\psi\rangle\langle\psi|\Big) =$

$\langle\psi|\mathcal{N}^{[0]*}(O_1^{[0]})\mathcal{N}^{[j]*}(O_2^{[j]})|\psi\rangle$, whereas the $n \geq 2$ observables $\mathrm{tr}\left(\rho^n O_1^{[0]} O_2^{[j]}\right) = \mathrm{tr}\left(\mathcal{N}^{\otimes n}((|\psi\rangle\langle\psi|)^{\otimes n}) d_n O_1^{[0]} O_2^{[j]}\right) = \langle\psi^{\otimes n}|\mathcal{N}^{*\otimes n}(d_n O_1^{[0]} O_2^{[j]})|\psi^{\otimes n}\rangle$, which involves a string operator as $\mathcal{N}^{*\otimes n}(d_n)$ is a product of the twist operators on all sites, $d_n = \otimes_j d_n^{[j]}$. The string operator then corresponds to a defect in the $n-$copied CFT.

For $n = 2$, we can further regard the density matrix as a state on the doubled Hilbert space $|\rho\rangle$. Then the purity and the Renyi correlator are the usual norm and correlation function of the doubled state, i.e., $\mathrm{tr}\,\rho^2 = \langle\rho|\rho\rangle$ and $\mathrm{tr}\left(\rho^2 O_1 O_2\right) = \langle\rho|O_1 O_2 \otimes I|\rho\rangle$. The evolution of the doubled state under local channels can be represented as a short-time imaginary time evolution, which renormalizes into a line defect inserted at imaginary time $d = 0$ slice, see Supplementary Figure 1. The Renyi correlator becomes a two-point correlation function on the defect, as shown in Supplementary Figure 1. Under the conformal transformation onto a cylinder with circumference L , one then expects that

$$C^{(2)}_{O_1O_2}(l) = A + B\left(\frac{L}{\pi}\sin\left(\frac{\pi l}{L}\right)\right)^{-\eta_2} \tag{S1}$$

where η_2 is twice the leading scaling dimension of the defect operator and A represents a possibly nonzero constant piece.

Below we study the Renyi correlators for the critical Ising model under X and Z dephasing numerically and extract the critical exponents η_2. We will find a number of new exponents that do not appear in the defect operator content of a single-copied Ising CFT, where the latter is thoroughly studied in Ref. [1]. This suggests that the decoherence corresponds to more intricate non-factorized defect of the double-copied Ising CFT, which is currently not fully classified.

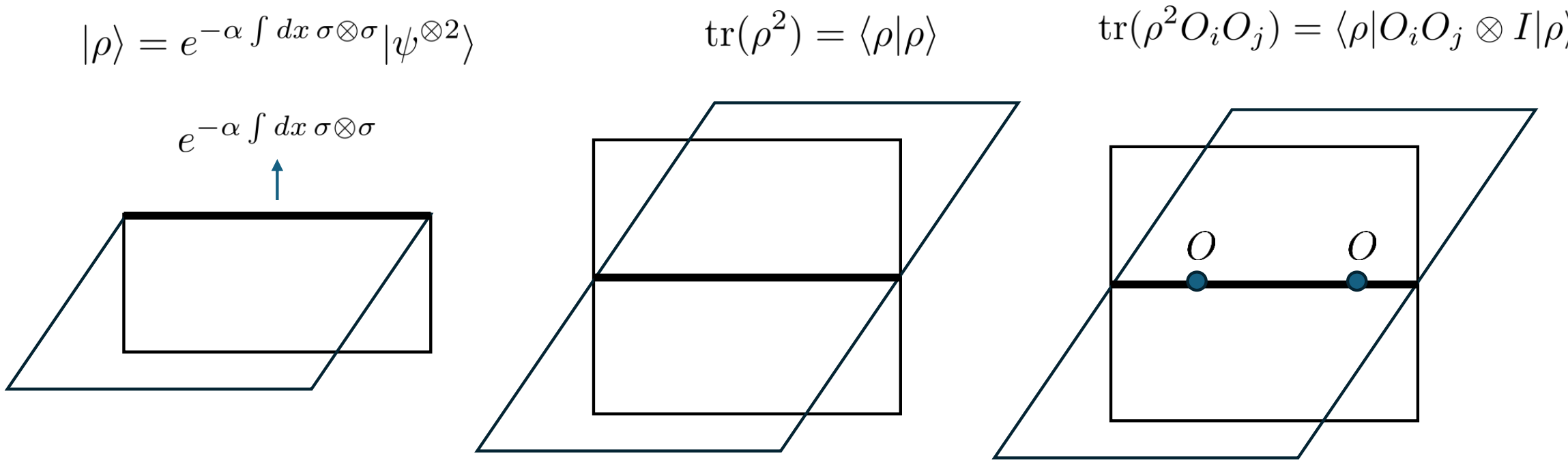

Supplementary Figure 1: Taking the critical transverse-field Ising model under X dephasing as an example, we illustrate the path integral representations of the doubled state $|\rho\rangle$ (left), the purity $\mathrm{tr}\left(\rho^2\right)$ (middle) and the Renyi correlator $\mathrm{tr}\left(\rho^2 O_i O_j\right)$ (right). Each rectangle represents the path integral on half plane, preparing one copy of the ground state $|\psi\rangle$. The thick line, inserted at the $d = 0$ slice, represents the defect that corresponds to the dephasing channel in the replicated CFT Ising$^{\otimes 2}$. The Renyi correlator corresponds to the two-point correlation function of defect operators.

X dephasing

We consider the Renyi correlators for X dephasing.

$$C^{(2)}_{XX}(l) = \mathrm{tr}\left(\rho^2 X_0 X_l\right)/\,\mathrm{tr}\left(\rho^2\right) \tag{S2}$$

The correlation function is long-ranged, see Supplementary Figure 2. This suggests that the function is of the form

$$C^{(2)}_{XX}(l) = A + B\left(\frac{L}{\pi}\sin\left(\frac{\pi l}{L}\right)\right)^{-\eta_2}, \tag{S3}$$

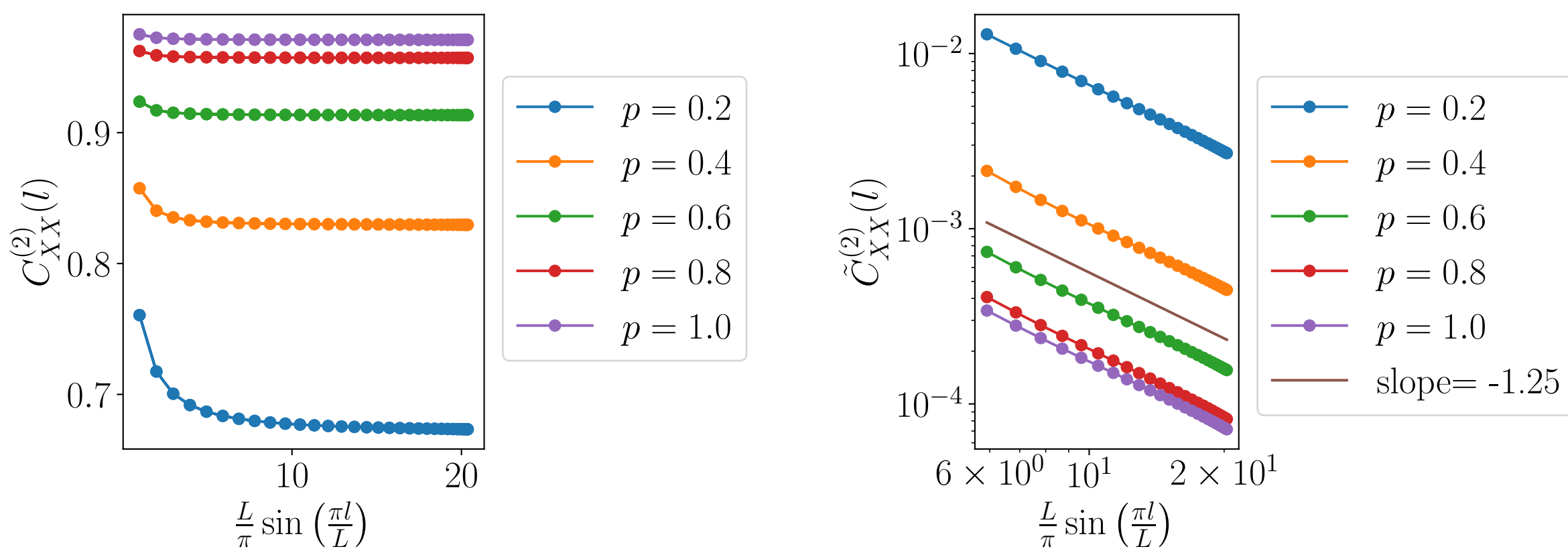

Supplementary Figure 2: Renyi-2 XX correlator $C^{(2)}_{XX}(l)$ of the transverse field Ising ground state under X dephasing. For any $0 < p \leq 1$ the correlator saturates to a constant at large l (left). After subtracting the constant part, the tail is a power law decay with an exponent $\eta_2 \approx 1.25(5)$ (right).

where η_2 is twice the scaling dimension of a defect operator. In order to compute η_2, we denote

$$\tilde{C}^{(2)}_{XX}(l) = C^{(2)}_{XX} - A. \tag{S4}$$

We use the linear fit in logarithmic scale

$$\log\left(C^{(2)}_{XX}(l) - A\right) = -\eta_2 \log\left(\frac{L}{\pi}\sin\left(\frac{\pi l}{L}\right)\right) + \log B \tag{S5}$$

such that the slope corresponds to $-\eta_2$. Note that A is not universal. We determine A numerically such that the linear fit agrees with the numerical data best. The result is shown in Supplementary Figure 2. We find that $\eta_2 \approx 1.25(5)$ independent of p.

Next we consider the Renyi-2 ZZ correlator

$$C^{(2)}_{ZZ}(l) = \frac{\mathrm{tr}\left(\rho^2 Z_0 Z_l\right)}{\mathrm{tr}(\rho^2)}. \tag{S6}$$

This again saturates to a constant at long distance, see Supplementary Figure 3. We again find that $C^{(2)}_{ZZ}(l)$ has the same form, which is a constant piece A plus a power-law decaying piece $\tilde{C}^{(2)}_{ZZ}(l)$. We also numerically find that

$$A = \left(\frac{\mathrm{tr}\left(\rho^2 Z_0\right)}{\mathrm{tr}(\rho^2)}\right)^2, \quad \text{for } ZZ \text{ corrlelator} \tag{S7}$$

which gives

$$\tilde{C}^{(2)}_{ZZ}(l) = \frac{\mathrm{tr}\left(\rho^2 Z_0 Z_l\right)}{\mathrm{tr}(\rho^2)} - \frac{\mathrm{tr}\left(\rho^2 Z_0\right)}{\mathrm{tr}(\rho^2)} \frac{\mathrm{tr}\left(\rho^2 Z_l\right)}{\mathrm{tr}(\rho^2)} \tag{S8}$$

The connected correlator decays as

$$\tilde{C}^{(2)}_{ZZ}(l) = B\left(\frac{L}{\pi}\sin\left(\frac{\pi l}{L}\right)\right)^{-\eta_2} \tag{S9}$$

where we fit $\eta_2 \approx 3.5(2)$ for $p = 0.6$ and 0.8. The exponent fitted with $p = 0.2$ and $p = 0.4$ is drifting towards η_2 at large l as result of the RG flow of the channel towards the $p = 1$ fixed point.

Previously, it was conjectured [2] that the defect is factorized into $(|+\rangle\langle+|)^{\otimes 2} + (|-\rangle\langle-|)^{\otimes 2}$, where $|+\rangle$ and $|-\rangle$

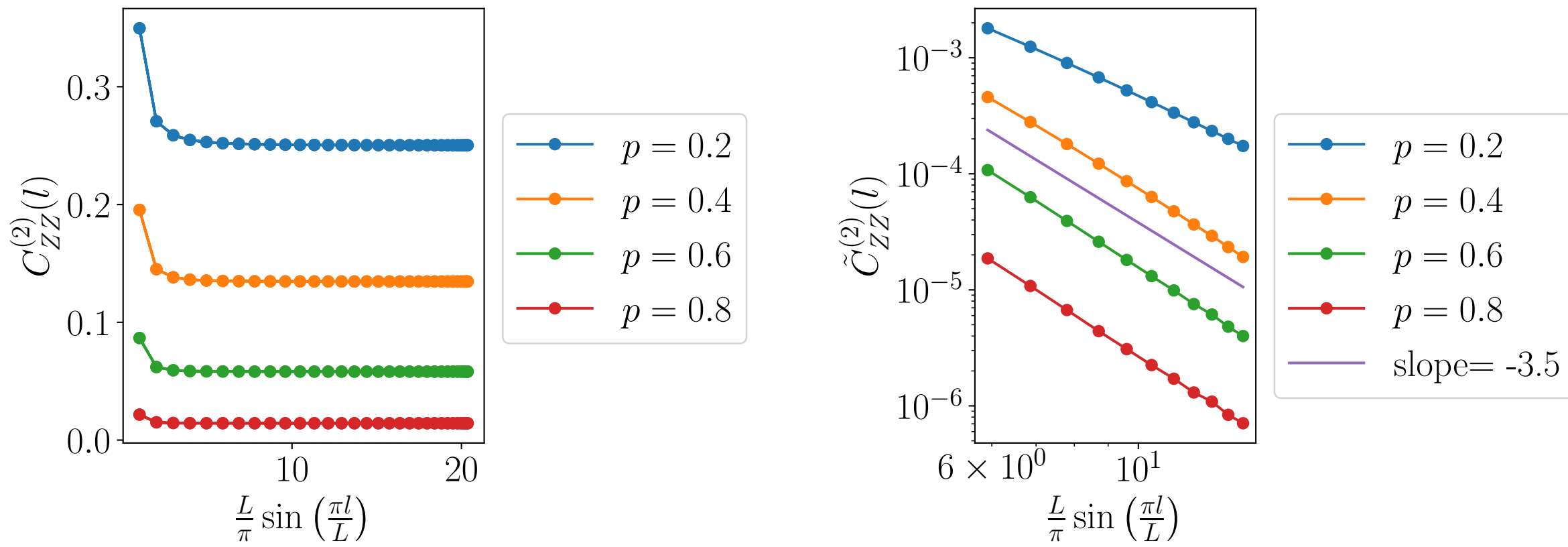

Supplementary Figure 3: Renyi-2 ZZ correlator $C^{(2)}_{ZZ}(l)$ of the transverse field Ising ground state under X dephasing. For any $0 < p < 1$ the correlator saturates to a constant at large l (left). After subtracting the constant part, the tail is a power law decay with an exponent $\eta_2 \approx 3.5(2)$ (right).

are the boundary state of the fixed boundary conditions in the Ising model. It was shown that this gives the correct subleading term in the Renyi entropy $S^{(n)}(\rho_A) := \frac{1}{1-n}\log \operatorname{tr}(\rho_A^n)$, for both A being the whole system and an interval. Naively, this factorization predicts that $\eta_2 = 4$, which equals the surface critical exponent of the extraordinary boundary phase transition of the Ising model. However, our numerical estimations of η_2 for both Renyi XX and ZZ correlators are far from 4.

Z dephasing

We consider the Renyi-2 XX and ZZ correlators for the state under Z dephasing. The XX correlator decays as a power law

$$C^{(2)}_{XX}(l) = B\left(\frac{L}{\pi}\sin\left(\frac{\pi l}{L}\right)\right)^{-\eta_2} \tag{S10}$$

where η_2 continuously varies with the noise rate p. See Supplementary Figure 3. Also, $\eta_2 = 2\Delta_\sigma = 1/4$ for $p = 0$ and increases with p. The numerics indicate that $\eta_2 \to 1.0$ as $p \to 1$.

The Renyi-2 ZZ correlator again saturates to a constant at long-distances. We consider the connected correlator $\tilde{C}^{(2)}_{ZZ}(l)$, where the constant piece has been subtracted, and find a power-law decay,

$$\tilde{C}^{(2)}_{ZZ}(l) = B\left(\frac{L}{\pi}\sin\left(\frac{\pi l}{L}\right)\right)^{-\eta_2} \tag{S11}$$

where we fit the slope $\eta_2 \approx 2.0(1)$ for small p ($p \leq 0.7$) using the range of $6 \leq l \leq 16$ for the $L = 64$ spin chain. For larger p, we also fit that $\eta_2 \approx 2.8(1)$ for $p = 0.8$ and $\eta_2 \approx 3.3(1)$ for $p = 0.9$. However, the slope is drifting to larger values as one fits with larger l, indicating that the drifting is a finite-size effect.

To summarize, we have the following table (Supplementary Table 1).

Optimal fidelity of decoding at small sizes

Here we show the optimal entanglement fidelity computed from the SVD decoder optimization (described in Methods) for X and Z dephasings. The result is shown in Supplementary Figure 6. For Z dephasing, F_e increases with L for constant p, indicating a finite decoding threshold. For X dephasing, we plot the horizontal axis as $\tilde{p} = pL^{\nu}$ with $\nu = 1/2, 3/4$. Comparing curves for different L shows the change of F_e when we choose $p \propto L^{-\nu}$. The perturbation

	Constant piece A	Exponent, η_2
$C^{(2)}_{XX}$, X dephasing	Nonzero	1.25(5)
$C^{(2)}_{ZZ}$, X dephasing	$(C^{(2)}_Z)^2$	3.5(2)
$C^{(2)}_{XX}$, Z dephasing	0	$0.25 \leq \eta_2 \leq 1.00(5)$
$C^{(2)}_{ZZ}$, Z dephasing ($p \leq 0.7$)	$(C^{(2)}_Z)^2$	2.0(1)

Supplementary Table 1: The constant part and the exponent of the Renyi correlators, where $C^{(2)}_Z := \mathrm{tr}(\rho^2 Z_0)/\,\mathrm{tr}(\rho^2)$. The naive expectation that the X dephasing induces a factorized defect suggests that first two exponents $\eta_2 = 4$. This expectation is, however, ruled out by the numerical simulation. The field-theoretic explanation of these exponents remains open.

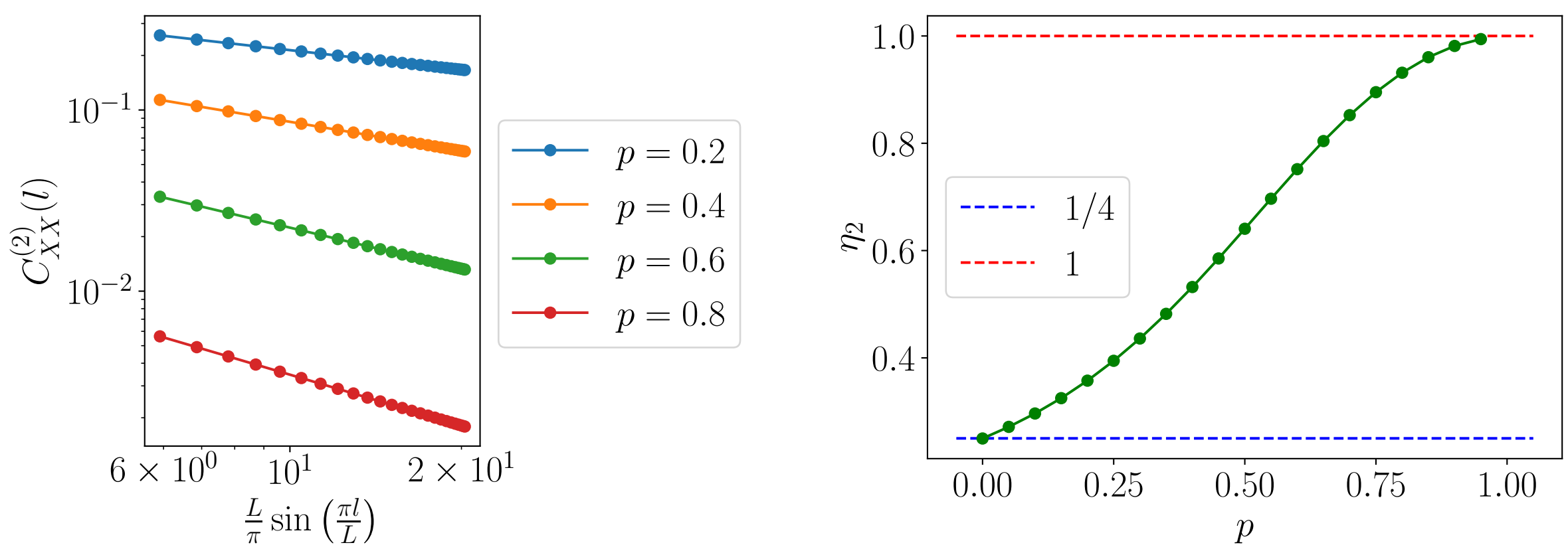

Supplementary Figure 4: Renyi-2 XX correlator $C^{(2)}_{XX}(l)$ of the transverse field Ising ground state under Z dephasing. The correlator decays as a power law, with exponent η_2 increasing with p (left). The numerical value is $\eta_2 = 1/4$ at $p = 0$ and $\eta_2 \to 1.00(5)$ as $p \to 1$ (right).

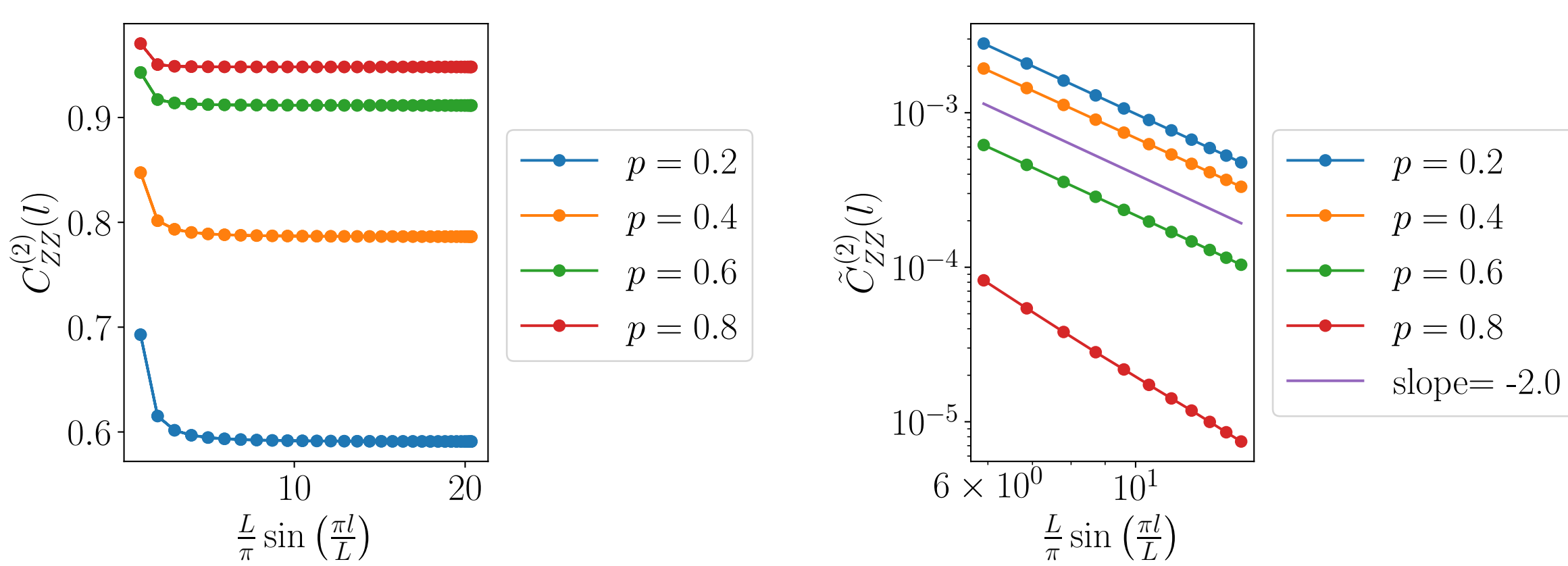

Supplementary Figure 5: Renyi-2 ZZ correlator $C^{(2)}_{ZZ}(l)$ of the transverse field Ising ground state under Z dephasing. The correlator saturates to a constant at large l (left). After subtracting the constant part, the tail is a power law decay with an exponent $\eta_2 \approx 2.0(1)$ for $p \leq 0.6$ (right). For $p = 0.8$, the fit with $6 \leq l \leq 16$ gives that $\eta_2 \approx 2.8(1)$, which drifts towards larger values for larger l. This can be seen as a finite-size effect.

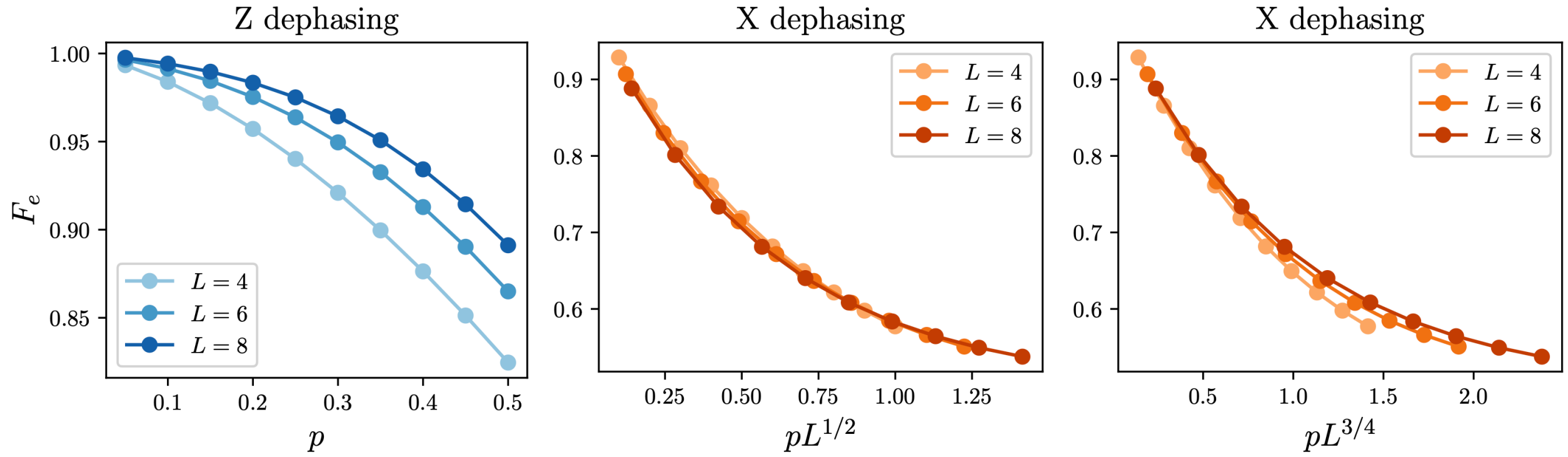

Supplementary Figure 6: Comparing the Z and X dephasing under different noise rates (from left to right: constant p, $p \propto L^{-1/2}$, $p \propto L^{-3/4}$). For Z dephasing and constant p, one finds that F_e increases with system size. For X dephasing, the decoder fails to correct the error rate at $p \propto L^{-1/2}$ but can correct a subextensive dephasing error rate at $p \propto L^{-3/4}$.

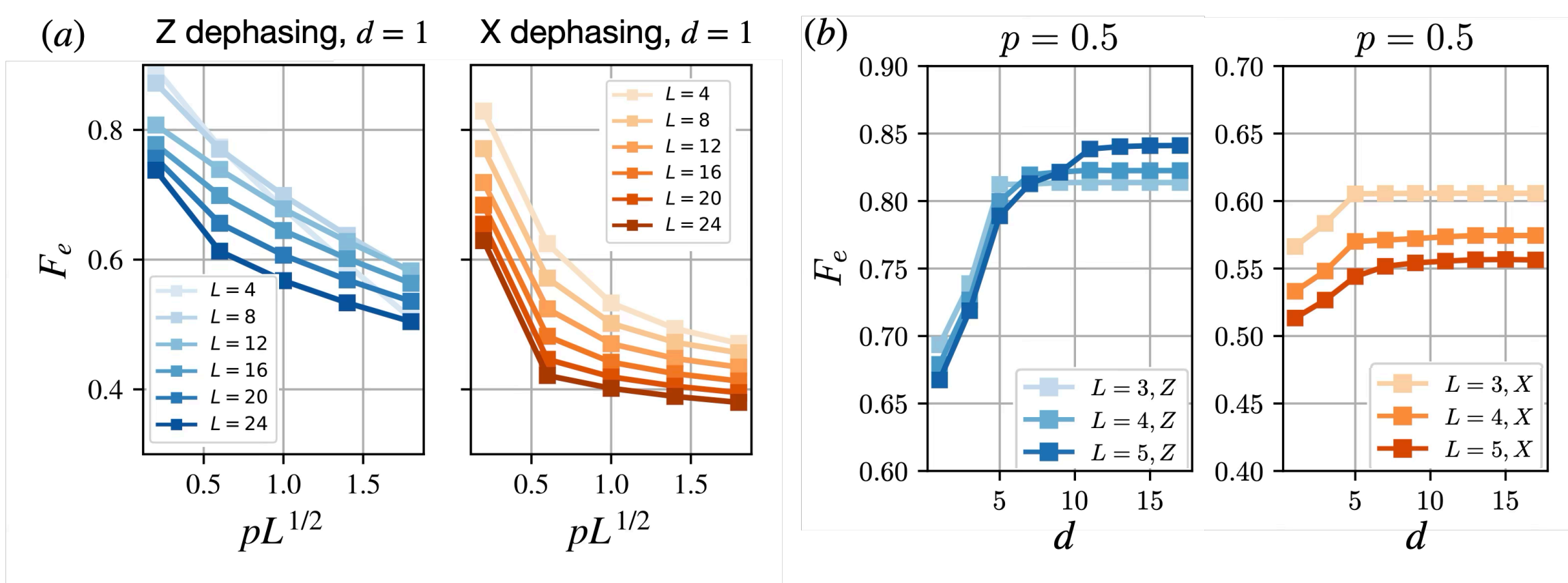

Supplementary Figure 7: (a) Entanglement fidelity obtained by a decoding circuit of depth $d = 1$, contrasted with Fig. 4 of the main text. At depth 1, the variational decoder already distinguishes the two channels at $L = 4, 8$ but struggles at larger system sizes $L \geq 12$. (b) For small system sizes $L = 3, 4, 5$, we test the optimal decoding depths.

theory in Ref. [3] suggests that F_e increases with L if $\nu > 1 - 2\Delta_\sigma = 3/4$ and decreases with L if $\nu < 3/4$. This prediction holds in the limit $p \to 0$ and $L \to \infty$. For small system sizes $L = 4, 6, 8$, we find that F_e slightly decreases with L for $\nu = 1/2$ and slightly increases with L for $\nu = 3/4$. Therefore, we choose the noise scaling with $\nu = 1/2$ to distinguish between Z and X dephasing.

Additional variational decoding results

The numerical methodology underlying the variational decoder (MPS construction of the CFT codeword, gradient-based optimization with the warm-start strategy, and transfer learning across system size) is described in Methods. Here we present additional decoding results that complement Fig. 4 of the main text.

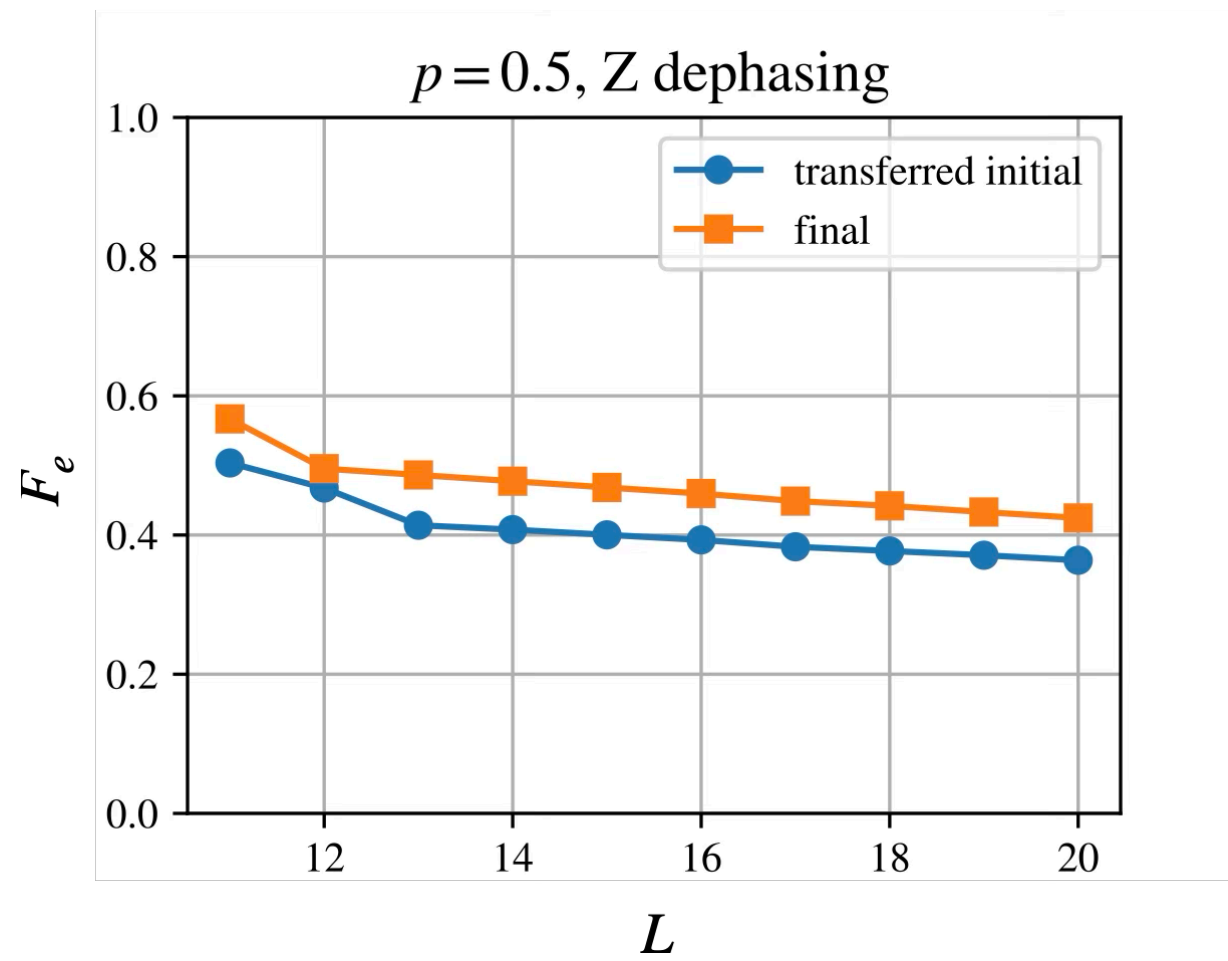

Supplementary Figure 8: Test performance of the transfer learning of a depth-1 decoder of the Ising CFT code with $p = 0.5$ Z dephasing (see Methods for the algorithm). The figure shows decoding fidelity F_e as a function of system size L, comparing transferred initialization (*initial*) with post-optimization results (*final*); the initial curve is consistently high over $L = 11$–20, showing the transferred parameters serve as a good initialization, with significant improvement over random initializations and enabling efficient measurement in experiments.

Depth-1 decoder at additional system sizes

Transfer learning performance

We observe consistently high initial F_e that avoids the exponential decay associated with random initialization, demonstrating the effectiveness of the transfer-learning strategy described in Methods.

Variational decoding for symmetry-protected topological phases

To show the versatility of the variational decoder, we test it on another class of physically-inspired QECC associated with symmetry-protected topological (SPT) phases. A 1D SPT phase with open boundaries naturally defines a QEC code: the quasi-degenerate edge states encode logical qubits. The code distance with respect to symmetry-preserving local errors is linear in the system sizes, and this protection persists under symmetry-preserving transverse-field deformations within the phase [4, 5]. Correspondingly, the SPT phase is also robust under non-maximal strongly symmetric dephasing, where the mixed state can be shown to be two-way connected to the pure SPT state under local symmetric channels [6, 7]. Under non-symmetric noise, the SPT phase is no longer robust, becoming a trivial phase or an average SPT [8, 9]. Correspondingly, the QECC is not robust against uniform non-symmetric noise because the noise has nonzero overlap with the logical operator. Therefore, decodability of the logical information encoded in the edge modes also signals different mixed-state phases. Below, we show that the variational decoding scheme can be used to distinguish the SPT phases under symmetric or non-symmetric noises, focusing on the cluster state as an example.

Exact decoder at zero transverse field

We first study the decoder for the $\mathbf{Z}_2 \times \mathbf{Z}_2$ SPT under symmetric noise at the fix point. There are two logical qubits encoded into the 4-fold degenerate ground states with open boundaries. Consider the Hamiltonian of the cluster state on even number of sites with open boundaries,

$$H_c = -\sum_{i=1}^{N-2} X_i Z_{i+1} X_{i+2}. \tag{S12}$$

The two $\mathbf{Z}_2$ symmetries are generated by $Z_{\text{even}} = \prod_i Z_{2i}$ and $Z_{\text{odd}} = \prod_i Z_{2i-1}$, leading to the 4-fold degeneracy. Numerically, one could split the four degeneracy by introducing an exact boundary field,

$$H = H_c - g_1 X_1 - g_N X_N. \tag{S13}$$

Note that $[X_1, H_c] = 0$ and $[X_N, H_c] = 0$ so the eigenstates do not change. Taking the ground state of H by fixing g_1 and g_N to be positive or negative, one could obtain the four ground states $|\psi_{+,+}\rangle, |\psi_{+,-}\rangle, |\psi_{-,+}\rangle, |\psi_{-,-}\rangle$ of H_c. We focus on one of the two logical qubits by considering the subspace spanned by $\{|\psi_{+,\pm}\rangle\}$. In this subspace, one logical operator $X_1 = +1$ is fixed and there is one logical qubit remaining, with the logical operators being

$$\bar{Z} = X_{N-1} Z_N, \qquad \bar{X} = X_N. \tag{S14}$$

The entanglement fidelity calculation introduces a reference qubit R that gives the initial state

$$|\psi\rangle_{RQ} = \frac{1}{\sqrt{2}} \sum_{\alpha \in \{\pm\}} |\alpha\rangle_R |\psi_{+,\alpha}\rangle_Q. \tag{S15}$$

We consider a simple noise model acting only on even sites,

$$\mathcal{N} = \prod_{i=2}^{N/2} \mathcal{N}_{2i}. \tag{S16}$$

Note that the noise starts at $i = 2$ (site 4), which will be useful to find a simple decoder below. For X noise with $p = O(1)$, since the Kraus operators have $O(1)$ overlap with the logical operator $\bar{X} = X_N$, the noise is not decodable. For Z noise, it is exactly decodable through the following sequential channel:

$$\mathcal{D} = \mathcal{D}_{N-2,N-1,N}\, \mathcal{D}_{N-4,N-3,N-2} \cdots \mathcal{D}_{2,3,4}, \tag{S17}$$

where $\mathcal{D}_{2i,2i+1,2i+2}$ is a measurement-feedback channel by first measuring the stabilizer $S_{2i} := X_{2i} Z_{2i+1} X_{2i+2}$ and then applying Z_{2i+2} if the measurement outcome is -1. This corrects each error sequentially starting from site 4. As a result, the entanglement fidelity is exactly one,

$$F_e := \langle \psi_{RQ} | \mathcal{D}\mathcal{N}(|\psi_{RQ}\rangle\langle\psi_{RQ}|) | \psi_{RQ}\rangle = 1. \tag{S18}$$

Finally, in order to implement the decoding channel as a circuit, we derive the unitary Steinspring dilation of the channel,

$$\mathcal{D}_{2i,2i+1,2i+2}(\rho) = \mathrm{tr}_{A_{2i}} \left[U_{QA} \left(\rho_Q \otimes |0\rangle\langle 0|_A \right) U_{QA}^\dagger \right]. \tag{S19}$$

The action of the unitary U_{QA} can be derived from the Kraus representation

$$\mathcal{D}_{2i,2i+1,2i+2}(\rho) = \sum_{\alpha \in \{\pm\}} K_\alpha \rho K_\alpha^\dagger, \tag{S20}$$

where

$$K_+ = \frac{1 + X_{2i} Z_{2i+1} X_{2i+2}}{2}, \qquad K_- = Z_{2i+2} \frac{1 - X_{2i} Z_{2i+1} X_{2i+2}}{2}. \tag{S21}$$

The unitary dilation is then given by

$$U_{QA}(|\psi\rangle_Q |0\rangle_A) = \sum_{\alpha \in \{\pm\}} \left(K_\alpha |\psi\rangle_Q \right) \otimes |\alpha\rangle_A \tag{S22}$$

for all $|\psi\rangle_Q$ on sites $2i, 2i+1, 2i+2$. There is freedom in choosing how U_{QA} acts on $|1\rangle_A$, as long as unitarity is preserved.

In sum, we have constructed a decoding circuit with one layer of sequential circuit that corrects Z dephasing on even sites starting at site 4. Note that if the error is also allowed to act on site 2, the simple decoder would not

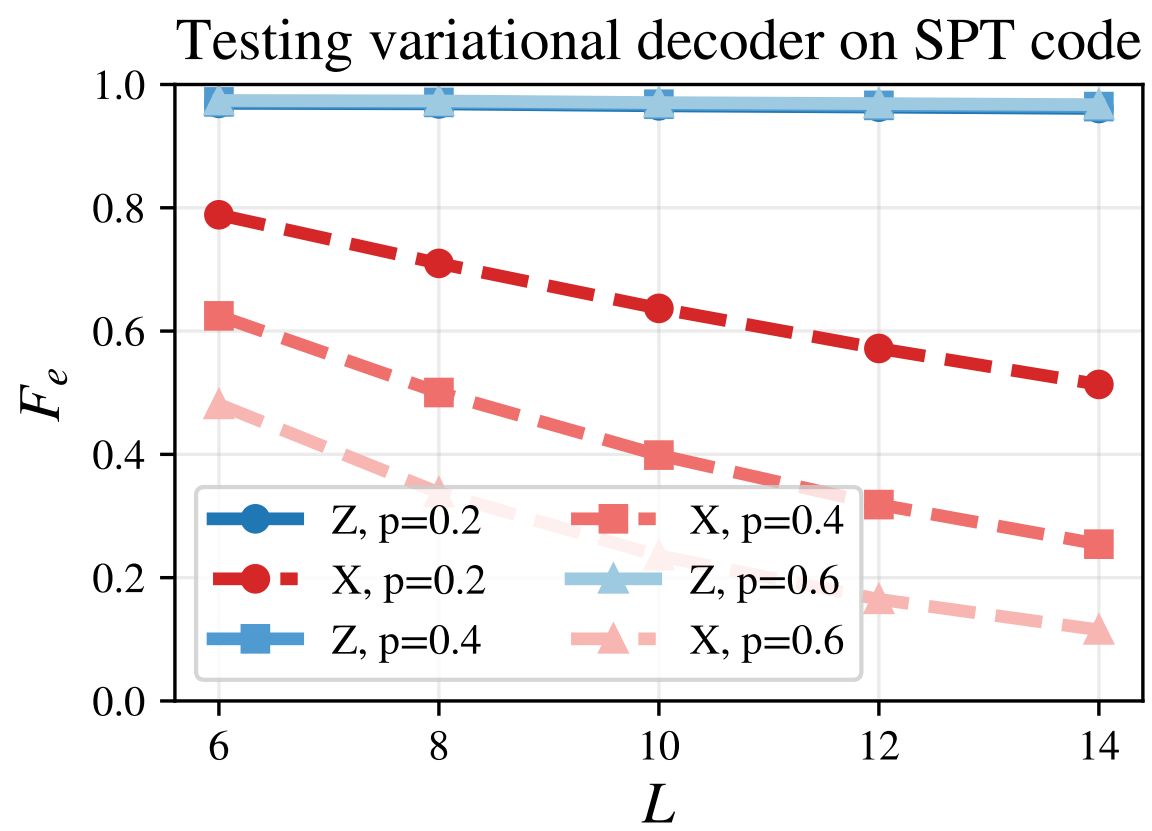

Supplementary Figure 9: Optimized entanglement fidelity versus system size L at $h = 0.2$ for the variational decoder initialized by the exact channel, showing robust high-fidelity recovery for Z noise (blue) and a strong fidelity drop with increasing L and p for X noise (red).

work, and a minimal-weight perfect matching decoder is required. As shown in Ref. [3], such a decoder can also be truncated to a local channel, with decoding error decaying exponentially with the locality of the channel.

Variational decoding at nonzero transverse field

We consider adding in transverse field h to the Hamiltonian of the cluster state, giving rise to

$$H_h = -\sum_{i=1}^{N-2} X_i Z_{i+1} X_{i+2} - h \sum_{i=1}^{N} Z_i, \tag{S23}$$

where the system remains in the SPT phase if $0 \leq h < 1$. At $h = 1$, the system undergoes an SPT-trivial phase transition described by the free compactified boson CFT [10]. In the SPT phase, the ground state is still 4-fold degenerate, with splitting of energy that decays exponentially with the system size N. While the exact decoder only works at $h = 0$, the shallow variational decoder is still expected to work in the entire SPT phase. Below, we test the performance of the variational decoding with $h = 0.2$ and see it efficiently distinguishes the correctable Z noise versus the uncorrectable X noise.

In numerics, the codeword states are generated by DMRG as MPS, then used to build the state $|\psi\rangle_{RQ}$ for entanglement fidelity. We apply local Pauli noise on even sites starting from site 4, and optimize a variational decoder of the sequential structure with one layer. The variational gates act on three physical qubits and three ancilla qubits and are initialized according to the exact decoder Eq. (S22) with small random perturbations. For numerical efficiency, we again choose two degenerate ground states, by introducing a small $g_1 X_1$ field on the boundary to lift the degeneracy

$$H = -\sum_{i=1}^{L-2} X_i Z_{i+1} X_{i+2} - g_1 X_1 - h \sum_{i} Z_i, \tag{S24}$$

One then uses the two lowest states (now exponentially split in energy) as the code subspace and use the exact decoder above as initialization for a shallow variational decoder.

For $h = 0.2$, the variational decoding fidelity F_e is shown in Supplementary Figure 9. For all p's, the fidelity for Z-noise stays high (between 0.96 and 0.98) and is only weakly size-dependent, remaining close to 1 even at larger L. This indicates that the exact-decoder structure remains effective in the SPT phase after variational refinement. In contrast, the fidelity for X-noise decrease strongly with both L and p, showing that this noise channel is not correctable in this setup. In summary, our result shows that the variational decoding approach is efficient in distinguishing different mixed-state phases obtained by SPT states under symmetric versus non-symmetric noises, even when the decoder is not known exactly.

Experimental details

Quantinuum H1 Processor

The Quantinuum System Model H1 is a trapped-ion quantum computer featuring 20 physical qubits arranged in a linear chain. Utilizing a quantum charge-coupled device (QCCD) architecture, it enables all-to-all qubit connectivity by transporting ions between five parallel interaction zones, allowing for flexible and efficient gate operations. The system supports a native gate set that includes single-qubit rotations, two-qubit ZZ gates, and arbitrary-angle ZZ gates. Notably, it offers mid-circuit measurement and qubit reuse capabilities, facilitating advanced quantum algorithms and error correction protocols [11, 12].

The H1 achieves high operational fidelity, with single-qubit gate infidelities as low as 1×10^{-5} and two-qubit gate infidelities around 1×10^{-3}. State preparation and measurement (SPAM) errors are typically around 3×10^{-3}, and memory errors per qubit per depth-1 circuit are approximately 2×10^{-4}.

Error analysis. Based on the above specs, the dominating error factor comes from entangling gates and SPAM error. For the Renyi-correlator experiment at $L = 6$, we use a total of 15 native two-qubit gates for preparing the state, and for each qubit: 3 measurements are implemented for the randomness in applying dephasing channel and randomized twirling, and another measurement is required for the final outcome; for the SPAM error along, this amounts to a total of $6 \times 4 \times 3 \times 10^{-3} \approx 7.2 \times 10^{-2}$ infidelity. Together with two qubit gate errors, a $\sim 9\%$ infidelity is expected in the final correlator outcome.

For decoding of the $L = 8$ CFT code, SPAM error is less significant there is no random Pauli applied before the measurement. we use a total of 93 entangling gates: 8 SU(4) gates for each encoding and decoding, additional 15 gates for the decoder; each SU(4) gates can be decomposed into 3 native ZZ gates; the overall estimated infidelity is at a little over 10%, matching the observed deviation in Fig. 5.

Next, we give an estimation on the noise rate in order to experimentally observe an advantage for $L = 24$ over $L = 8$ at decoding depth $d = 3$. We numerically observe that at $L = 24$ encoding $U_{\mathcal{E}}$ (and thus $U_{\mathcal{E}}^{\dagger}$) can be done with a relatively shallow depth, requiring 48 SU(4) gates; decoding at $d = 3$ amounts to 141 SU(4) gates. The whole circuit totals at 711 native two qubit gates with a straightforward Cartan decomposition. Therefore, it would suffice to have the each gate infidelity below 1×10^{-4}, which under the current progress of trapped-ion processors, considered achievable in the next 5 years [13]. Another remark is that, under the current compilation method to implement random Pauli twirling, Renyi correlator is more sensitive to SPAM errors than entanglement fidelity.

Implementation of quantum randomness

For implementing both the depolarizing channel and the randomized measurement, randomness plays a crucial role. However, QSAM does not have a built-in function for generating random numbers in real time. In theory, one could pre-generate all possible randomized circuits and submit them in advance. However, in practice, this approach introduces significant overhead in input size and increases quantum resource usage, as each quantum circuit submission incurs a fixed cost, regardless of the number of circuit executions.

To address this issue, we utilize extra ancilla qubits along with classical feedback as a natural quantum source of randomness. To implement a Pauli depahsing channel on a physical qubit, we apply a single-qubit rotation $R_X(\theta)$ to an ancilla qubit, where $\theta = 2 \arcsin\left(\sqrt{p/2}\right)$. Upon measuring the ancilla qubit, if the outcome is 1, a corresponding Pauli gate is applied to the system qubit. Since the QCCD architecture supports mid-circuit measurement and qubit reuse [11], a single ancilla qubit is sufficient for the entire physical system.

Realizing randomized Pauli measurements is slightly more complicated, as we need to select one of the three measurement bases (X, Y, or Z) with equal probability while relying only on qubits instead of qutrits. This can be achieved using two ancilla qubits initialized as 00. Specifically, we define measurement outcomes 00 and 01 as corresponding to the Z basis, 10 to the X basis, and 11 to the Y basis. The key trick is to apply rotations with different angles: we apply $R_X(2 \arcsin\left(\sqrt{2/3}\right))$ to the first ancilla qubit and a Hadamard gate to the second. It is straightforward to verify that the resulting probabilities satisfy $p_{00} = p_{01} = 1/6$ and $p_{10} = p_{11} = 1/3$, ensuring the desired probabilities of the basis selection.

This method effectively integrates randomness generation into the quantum circuit itself, reducing classical pre-processing overhead while maintaining efficient quantum resource utilization.

Classical shadow estimation of Renyi negativity

Another previously proposed probe of the mixed-state phases is the Rényi negativity. One such quantity is the Renyi negativity. Given a L-qubit, bi-partite quantum system, AB, consisting of $|A| = l$ and $|B| = L - l$ spins respectively, the n-th order Renyi negativity is defined as the following:

$$\mathcal{N}_A^{(n)} = \frac{1}{1-n}\log[\mathrm{Tr}(\rho^{T_A})^n/\mathrm{Tr}(\rho^n)]$$

. Without dephasing, the Renyi negativity satisfies the logarithmic scaling,

$$N_A^{(3)} = \alpha \log\left(\frac{L}{\pi}\sin\frac{\pi l}{L}\right) + O(1), \tag{S25}$$

For the Ising model considered in this work, the Renyi negativity shows distinct features for X, Y, and Z dephasings. The Renyi negativity becomes area law for X dephasing, indicating a relevant perturbation. For Z dephasing, the Renyi negativity is still logarithmic, but the coefficient α continuously decreases with p, indicating a marginal deformation. For Y dephasing, the Renyi negativity stays as the same logarithmic form with $\alpha = 2c/9 = 1/9$. One drawback of this quantity is that it is only nontrivial starting from Renyi index $n \geq 3$. For $n = 2$, even though the mixed-state phases are expected to be the same, the Renyi negativity does directly probe the phases.

The quantity can also be experimentally accessed with the random measurement method [14], which we briefly review here. For each copy of the state, we first perform randomized measurement by applying random local unitaries $u_1 \otimes ...u_L$, each sampled i.i.d. from a 3-design, and record the classical measurement outcomes $\boldsymbol{k} = \{k_1, ...k_N\}$. We repeat the the experiment M times and denote each set of measurement outcomes $\boldsymbol{k}^{(r)}$, from which we could construct an unbiased estimator:

$$\hat{\rho}_{AB}^{(r)} = \bigotimes_{i\in AB}\hat{\rho}_i^{(r)} = \bigotimes_{i\in AB}(3u_i^\dagger \left|\boldsymbol{k}_i^{(r)}\right\rangle\!\left\langle\boldsymbol{k}_i^{(r)}\right| u_i - \mathbb{I}) \tag{S26}$$

whose expectation gives raise to ρ_{AB} [15]. Next, the partially transposed (PT) moments, $p_n = \mathrm{Tr}[(\rho_{AB}^{T_A})^n]$ can be calculated:

$$\begin{aligned} p_n &= \frac{1}{n!}\binom{M}{n}^{-1}\sum_{r_1\neq r_2\neq r_3...\neq r_n}\mathrm{Tr}\Big\{\overrightarrow{\Pi}_A\overleftarrow{\Pi}_B\,\hat{\rho}_{AB}^{(r_1)}\otimes...\otimes\hat{\rho}_{AB}^{(r_n)}\Big\} &\text{(S27)}\\ &= \frac{1}{n!}\binom{M}{n}^{-1}\sum_{r_1\neq r_2\neq r_3...\neq r_n}\Pi_{i\in A}\,\mathrm{Tr}\Big\{\hat{\rho}_i^{(r_1),T}\otimes...\otimes\hat{\rho}_i^{(r_n),T}\Big\}\Pi_{i\in B}\,\mathrm{Tr}\Big\{\hat{\rho}_i^{(r_1)}\otimes...\otimes\hat{\rho}_i^{(r_n)}\Big\} &\text{(S28)}\end{aligned}$$

Notice that the condition $r_1 \neq r_2 \neq r_3... \neq r_n$ over the sum is necessary for guaranteeing the independence of the samples and forming an unbiased estimation. Naively, the classical computation time required is $O(M^n)$, which is still demanding, considering $M \sim 10^5$ in [14]. Luckily, one could further simplify Eq. S28 due to the symmetries in the equation. For example, for $n = 3$,

$$\begin{aligned} p_3 =& \frac{1}{3!}\binom{M}{3}^{-1}\mathrm{tr}\Big\{\sum_r[\hat{\rho}_{AB}^{(r),T_A}]^3 - 3\sum_r[\hat{\rho}_{AB}^{(r),T_A}]\sum_r[(\hat{\rho}_{AB}^{(r),T_A})^2])\Big\} &\text{(S29)}\\ &+ 2\,\mathrm{tr}\Big\{\sum_r[(\hat{\rho}_{AB}^{(r),T_A})^3])\Big\} &\text{(S30)}\end{aligned}$$

The experimental result for the $L = 6$, $p = 0.3$ result is demonstrated in Supplementary Figure 10. The difference between X and Z channels are clearly captured, in accordance with the theoretical expectation.

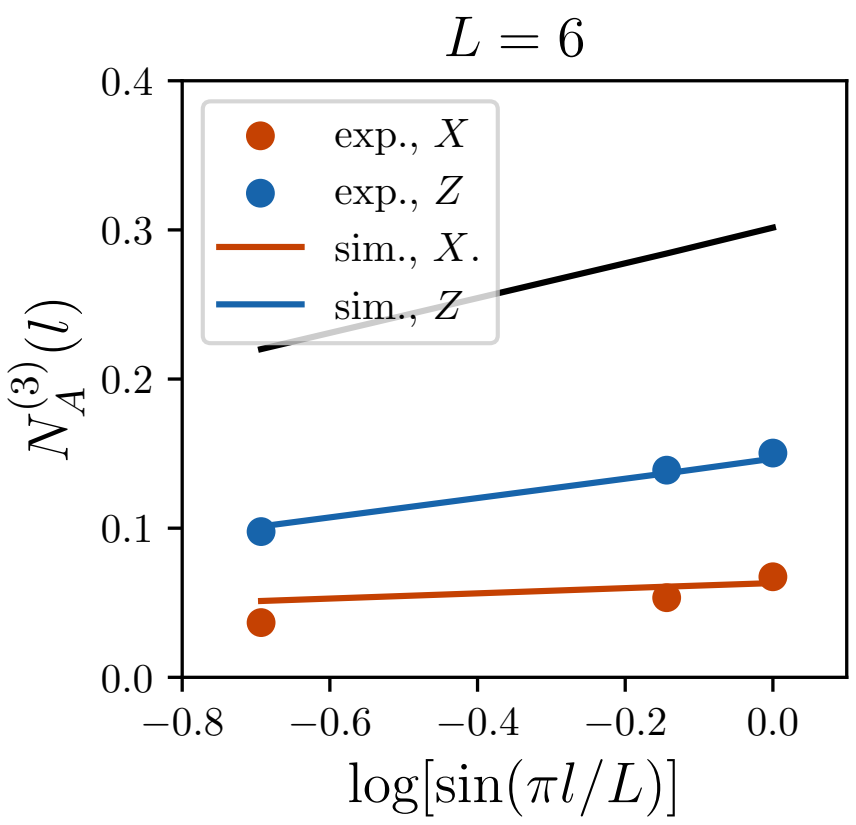

Supplementary Figure 10: The same experimental data set we use to compute Renyi correlator in the main text can be used to generate the $N_A^{(3)}$ here.

[1] M. Oshikawa and I. Affleck, Nuclear Physics B **495**, 533–582 (1997).
[2] R. Ma, Exploring critical systems under measurements and decoherence via keldysh field theory (2023), arXiv:2304.08277 [quant-ph].
[3] S. Sang, T. H. Hsieh, and Y. Zou, Physical Review Letters **133**, 210601 (2024).
[4] B. Zeng and D.-L. Zhou, Topological and error-correcting properties for symmetry-protected topological order (2014), arXiv:1407.3413 [quant-ph].
[5] D. V. Else, I. Schwarz, S. D. Bartlett, and A. C. Doherty, Phys. Rev. Lett. **108**, 240505 (2012), arXiv:1201.4877 [quant-ph].
[6] C. de Groot, A. Turzillo, and N. Schuch, Quantum **6**, 856 (2022).
[7] S. Sang, Y. Zou, and T. H. Hsieh, Physical Review X **14**, 031044 (2024).
[8] R. Ma and C. Wang, Physical Review X **13**, 031016 (2023).
[9] R. Ma, J.-H. Zhang, Z. Bi, M. Cheng, and C. Wang, Topological phases with average symmetries: the decohered, the disordered, and the intrinsic (2023), arXiv:2305.16399 [cond-mat.str-el].
[10] R. Verresen, R. Moessner, and F. Pollmann, Physical Review B **96**, 10.1103/physrevb.96.165124 (2017).
[11] J. M. Pino, J. M. Dreiling, C. Figgatt, J. P. Gaebler, S. A. Moses, M. Allman, C. Baldwin, M. Foss-Feig, D. Hayes, K. Mayer, *et al.*, Nature **592**, 209 (2021).
[12] M. DeCross, R. Haghshenas, M. Liu, E. Rinaldi, J. Gray, Y. Alexeev, C. H. Baldwin, J. P. Bartolotta, M. Bohn, E. Chertkov, *et al.*, arXiv preprint arXiv:2406.02501 (2024).
[13] A. Hughes, R. Srinivas, C. Löschnauer, H. Knaack, R. Matt, C. Ballance, M. Malinowski, T. Harty, and R. Sutherland, arXiv preprint arXiv:2510.17286 (2025).
[14] A. Elben, R. Kueng, H.-Y. R. Huang, R. van Bijnen, C. Kokail, M. Dalmonte, P. Calabrese, B. Kraus, J. Preskill, P. Zoller, *et al.*, Physical Review Letters **125**, 200501 (2020).
[15] H.-Y. Huang, R. Kueng, and J. Preskill, Nature Physics **16**, 1050 (2020).
