# Supplementary material for: Probing mixed-state phases on a quantum computer via Renyi correlators and variational decoding
Source: Nat Commun. 2026 Jun 27;17:8047. doi: 10.1038/s41467-026-73814-6 (PMC13454128; doi:10.1038/s41467-026-73814-6)
Supplement: Supplementary file 2 [file 41467_2026_73814_MOESM2_ESM.pdf]

nature portfolio

Peer Review File

Probing mixed-state phases on a quantum computer via Renyi correlators and variational decoding

Corresponding Author: Dr Yuxuan Zhang

This file contains all reviewer reports in order by version, followed by all author rebuttals in order by version.

A version of this paper was originally rejected for publication by Nature Communications, however that decision was reconsidered after appeal by the authors.

Version 0:

Reviewer comments:

Reviewer #1

(Remarks to the Author)
The submitted manuscript studies the effects of dephasing on the structure of a quantum state prepared in a quantum device. The authors focus on so-called Renyi correlators, and on the entanglement fidelity. These objects have been studied in many recent theoretical works aiming to classify quantum states generated by local decoherence. The experimental results (in Figs. 2 and 5) are the observations that (i) after X errors, the Renyi correlator decays more slowly with separation than in the initial state, (ii) after Z errors, it decays more quickly, (iii) the entanglement fidelity following Z errors is higher than after X errors.

The entanglement fidelity has a clear quantum information theoretic meaning, and the method for measuring it is novel. The Renyi correlator does not have such a clear meaning, and can only be extracted using exponential resources (as discussed by the authors). I appreciate that the Renyi correlator is of interest for the community of theorists actively working on classifying mixed state phases but here its investigation is not very well motivated. Here it comes across as one of (many) objects that can be constructed from shadows.

The experiment studies systems of up to eight qubits prepared in an approximation to the critical Ising ground state. However, the initial quantum state is not characterized. For example, in Fig. 2(b) no experimental data is shown for the case without dephasing, and no information is provided on how the state is prepared (aside from the fact that a variational quantum circuit is used). The authors' interpretation of their result hinges on this state having correlations resembling those of the true critical Ising ground state, so it seems necessary to at least show conventional correlation functions compared with the corresponding finite-size numerical results.

Should we believe that Ising CFT is a relevant framework for describing the effects of decoherence on six (or even eight) qubits?

In the abstract, the authors write that they use shadow tomography to observe a Renyi correlator in two distinct phases, one having power-law correlations and the other having long-range correlations. This is pretty unclear: numerical calculations in large systems show a significant difference in correlations, but it is not possible to say whether the experiment detects long-range correlations in one case and power-law correlations in the other: There are only three experimental data points for each data set in Fig. 2. It would be fair to say that the experiment detects e.g. 'a larger Renyi correlator' in one case.

In Fig. 1 it would be helpful if
1. The geometry of the experimental system is displayed. Is this a system with open boundary conditions, with correlations between two sites away from the ends of the chain, or between one end and a site in the middle?
2. There is a standard scale in Fig. 2(b), in units of sites. At present it is not clear what the data shows.
3. Finite size experimental results are compared with numerical predictions (shown as points) for the finite size chain.
At present, the information provided about Fig. 2(b) is insufficient to understand what is being displayed.

As a general point, the lines between interpretation, theory, and experimental results are not particularly clear. To improve

the clarity of the paper, it seems that essential that the captions of Figs. 3 and 4 state explicitly that these are numerical rather than experimental results.

The claim in the introduction that the initial state can be interpreted as a quantum error correcting code has the potential to be extremely confusing to readers who are not in the theoretical community actively working on these problems. In particular, the authors write ‘Traditional QEC codes, typically based on the stabilizer formalism and exemplified by topological codes, require implementation in at least two spatial dimensions. On the other hand, recent theoretical studies have shown that many physical states, even in one dimension, can be used as QEC codes’. This sentence creates the impression that the states studied here can be viewed as useful QEC codes. The reality is that QEC is a useful framework that can be applied to the study of mixed state phases. It seems important to maintain a sharp distinction between actual QEC, on the one hand, and CMT interpretations through the lens of QEC, on the other.

The line ‘they are typically characterized by nonlinear observables and information-theoretic quantities’ is likely to be confusing to readers outside outside of the theoretical community working on these problems. ‘Nonlinear’ in what?

There is a conjecture in the discussion that references numerical calculations that are not included in the manuscript: ‘Based on our numerical simulations on small sizes, we conjecture that optimally decoding a correctable dephasing error at a finite rate requires the circuit layer scaling as tau = O(L)…’. This leads to the possibility of detecting difference mixed state phases in polynomial cost. In connection with this, it would be very useful if the numerical results which are the basis for this conjecture are included.

In connection with this: Do the experimental decoding results in Fig. 5 use decoders constructed from numerics? If so, does this scheme not require either full state tomography at the start, or otherwise the preparation of a quantum state that is (in principle) known exactly?

The authors write that, with a decoder of unit depth, the experimental entanglement fidelity is already different for X and for Z errors. Is it reasonable to expect that a local circuit would be sufficient here, given that deep circuits were required for numerics?

The method for measuring F_e (used in Fig. 5) is currently illustrated in Fig. 1 but is not described in the main text. In practice the circuit used for initial state preparation, as well as its inverse, are subject to errors. When these errors are taken into account, is the entanglement fidelity that is eventually measured still a lower bound on the true entanglement fidelity?

In summary, the experimental results in this paper would benefit from more explicit explanations. Since the question of whether mixed state phases can be observed is essentially open, with papers even claiming that they cannot be observed directly in data (see arXiv:2504.12233), a proof of concept for such an experiment should provide a detailed analysis of the resource requirements. While the theoretical and numerical results are very interesting, these sit apart from the experimental results. That said, my overall view is that neither the theoretical nor experimental results (taken on their own) merit publication in Nature Communications.

(Remarks on code availability)

Reviewer #2

(Remarks to the Author)
In this manuscript, Zhang et al. experimentally studied mixed-state phases via Rényi correlators. The study was carried out on Quantinuum’s H1 processor. The authors claim to demonstrate characterization methods for mixed-state phases. Specifically, under controlled decoherence channels, the decoding capability of error-correcting codes using variational circuits was tested. The study is presented in two parts. First, Rényi correlators via classical shadows were used to distinguish mixed-state phases. The results are corroborated with conformal field theory (CFT) calculations. These findings were complemented by entanglement fidelity diagnostics. Second, the authors tested the decoding fidelity associated with error-correcting codes. I find the results overall satisfactory. I would like to emphasize that these diagnostic tools are essential for quantum simulation platforms, where distinct features of mixed-state phases can become important in studying universality classes of interactions.
Detailed Comments:
1)I wonder if these kinds of tests are more appropriate for larger system sizes and more complex quantum states, where full state tomography becomes impractical. In this study, the system size is relatively small—up to 8 qubits. While I still find the results promising, it is unclear to me what the key novelty of the presented case study is. A clearer articulation of the conceptual or methodological advancement, beyond demonstrating feasibility on a small system, would strengthen the manuscript.

2)The experimental error bars in Figures 2 and 3 appear to be unusually small. Could the authors clarify the measurement budget? Specifically, how many random unitaries were implemented, and how many shots per random unitary were taken? Additionally, what assumptions underlie the reported error bars? They mentioned Jackknifing, on what this Jacknifing was performed. Understanding this would help evaluate the statistical reliability of the results.

3)Did the authors characterize the intrinsic (physical) noise affecting their observations, beyond the engineered noise channels they introduced? For example, in the presence of engineered X-type noise, are there other background noise

sources that might contribute to discrepancies between theory and experiment? The manuscript briefly mentions gate infidelities—could these also influence the observed behavior? Furthermore, the authors introduce dephasing channels using additional random operations. Can these be reasonably modeled as white noise, which is often the assumption in theoretical calculations? It would be helpful to include a more detailed discussion of these aspects in the manuscript.

4)The manuscript should mention the system size clearly and early on. For instance, beginning with the third paragraph of the Introduction, a sentence such as: “In this work, we… on a trapped-ion quantum processor with N= 5-8 qubits” would help contextualize the scale of the experiment right away.

5)The Supplementary Material (SM) is difficult to follow without any sectional structure. It would be helpful if the SM were divided into clearly labeled subsections. Furthermore, references to the SM within the main text should include specific section numbers to improve navigation.

6)I was confused by the simulation results shown for L=64, especially considering the largest experimental system size used is only N=8. If conformal field theory (CFT) validation requires large system sizes, this should be explicitly stated and discussed in the manuscript to avoid ambiguity.

7)What is the motivation behind choosing the transverse-field Ising model for this study? Would similar behavior be expected for other Hamiltonians, or are the observed features specific to the ground state of the transverse-field Ising model? While I understand that ground state preparation is perhaps easy for this model, a brief justification would help clarify its role in the context of the study.

8)The authors study two noise channels separately. Could there be any nontrivial interplay if both noise channels act simultaneously? Would the current estimators be capable of distinguishing the corresponding mixed-state phases under such combined noise? Including a brief discussion or comment on this point will improve understanding the role of noises and their combined effects.

I find the overall results promising and potentially impactful, opening the door to several interesting research directions, as already pointed out in the outlook of the manuscript. In particular, bridging concepts from quantum error correction, quantum simulations, and conformal field theory offers a compelling and rich framework for future exploration. However, the presentation of the manuscript could be significantly improved for better readability. Key technical details—such as the measurement budget, error analysis, and specific information about the quantum system used—are insufficiently discussed. With a thorough revision that addresses these points, I believe the manuscript could be suitable for publication.

(Remarks on code availability)

Reviewer #3

(Remarks to the Author)
The manuscript by Zhang et al. reports on the experimental realization of mixed-state phases on the Quantinuum H1 quantum computer and the characterization of a corresponding CFT-based QEC code with the implementation of a decoder for small system sizes. More specifically, they prepare small (L = 4–8) ground states of the critical transverse-field Ising chain, and apply engineered X or Z dephasing, and then probe the resulting mixed-state phases with two complementary diagnostics: (i) Second-order Rényi two-point correlators, reconstructed with classical-shadow tomography and
(ii) a ladder-ansatz variational decoder that maximises entanglement fidelity Fe between the logical subspace and a reference qubit. Their theory shows that even with a single decoder layer, the hardware data already separates the two phases: Fe rises with L under Z noise but falls with L under X noise as predicted by their previous theory work
[PRL 133 210601 (2024)].

While this work is definitely interesting and worthy of publication, I do not think it hits the bar of novelty required for publication in Nature Communications for the following reasons:

1. While the manuscript convincingly demonstrates the first experimental probes of CFT-based mixed-state phases and implements the first working decoder for such a code, all the theory advances were already established in the recent paper PRL 133 210601 (2024) coauthored by two of the authors of this work. The novelties of this work are the implementation of the decoder and the numerical evidence, through a greedy algorithm, that finding the decoder variationally can be done in O(1) steps for small system sizes. My opinion is that this is a follow-up to their previous work, which doesn't add a significant degree of novelty to warrant publication in Nat. Comm., also because the experimental evidence, even if gathered for more system sizes, is not very convincing (see below points 3. and 4.).

2. The evidence gathered at small system sizes suggests that decoding is efficient, but only if the decoder does not have an exponential number of gates, and it is not clear when this condition occurs. The conjecture in the outlook that the number of layers in the decoder scales linearly with the system size is not well motivated. Also, it is not clear what they mean by “it grows much more slowly” in the uncorrelated dephasing phase: logarithmically? O(1)? Since this is one of the main takeaways of the paper, I think the authors show limited evidence for it.

3. About the experimental evidence, in Figure 4, it is not clear that the claim that the decoder performance for X-noise nearly saturates at \tau=5 for all three sizes is justified, especially for the L=5 case. Probably it is more like tau=15 in the L=5 case.

4. In Figure 5, the improvement from L=4 to L=8 at pL^1/2>~1 can be really seen only in the theory curves. If one looks at the error bars, all those data points seem statistically compatible, so it is a bit of a stretch to say that the authors verified experimentally that there is a region where the L=8 code outperforms the L=4 code. If there were no theoretical curve, one would not even have guessed that there is a crossing point around pL^1/2=1. So the claim that the authors experimentally verify this feature is a bit overstated.

5. The present code is not universal as it does not allow for corrections for X depolarizing noise (as already pointed out in the 2024 PRL). Therefore, from the application point of view is a bit limited.

Overall, this work seems a solid follow-up of the 2024 PRL, where small-scale experimental implementation has been realized (with some limitations) and decoding with a variational approach has been fleshed out. However, I don't think this is enough to warrant publication in Nature Communications.

Other Clarifications:
1. How would the results in Figures 2 and 3 change when p>0.3?
2. It is not clear in the first sentence of the last paragraph of the first column on page 5, what is ``d=1". Since D=2 throughout the paper, maybe it refers to dimensionality. Can the authors clarify?

(Remarks on code availability)

Reviewer #4

(Remarks to the Author)
I co-reviewed this manuscript with one of the reviewers who provided the listed reports. This is part of the Nature Communications initiative to facilitate training in peer review and to provide appropriate recognition for Early Career Researchers who co-review manuscripts.

(Remarks on code availability)

Version 1:

Reviewer comments:

Reviewer #1

(Remarks to the Author)
Thank for sending this article, which has improved since the initial submission. However, I have technical concerns about the analysis of the experimental data, and about the claimed efficiency of the variational decoding method. Overall, while the theoretical results are interesting, I still do not find that the experimental results or their analysis merit publication in Nature Communications.

In Fig. 2 the authors fit a power law to an experimental dataset consisting of just three points, and where neither the horizontal or vertical axis values change by anywhere close to one order of magnitude. It is difficult to take the results of this fit, and the corresponding exponents, seriously. This exponent is also quoted without an error bar.

In Fig. 3 the authors compare F_e to the channel distance d_rho, but how is d_rho determined? Are the dotted and dashed lines numerical calculations, or is d_rho determined using full tomography of the experimental state? If numerics, is it reasonable or even meaningful for the experimental result for F_e saturate the bound (this is mentioned below Eq. 9)? There is room for improvement in the presentation of this part of the manuscript.

In the final paragraph before the discussion, the authors write 'Compared with Renyi observales, which requires exp(O(L)) samples for shadow tomography [79], the sampling complexity for variational decoding is O(1).'

I do not understand how this can be the case in general. The sample complexity is only O(1) if the optimal decoder is known to extremely high accuracy. However, if the decoder differs from the optimal one by just a near-identity single-qubit unitary on each site, then estimating the fidelity in experiment requires exponential time.

The situation relevant to a typical experiment is where one starts from not knowing the decoder and uses the variational method to find it. But, to optimize the fidelity using a variational method, one needs to evaluate F_e for different variational parameters. Because F_e is generically exponentially small in L, the sample complexity required to experimentally measure F_e for a particular instance of the decoder is exponentially large in L. This would mean that it takes exponential time to optimize the decoder and then use this method (that is, if optimization is done for the experimentally prepared state).

If it is possible to successfully optimize using numerics, then by assumption the state prepared in experiment is easy to store

and study on a classical computer. This restricted situation would indeed give O(1) sample complexity in the experiment, but the applicability of the method is then extremely limited. Is an experimental method useful if it only works in settings where one has access to exact numerical simulations?

(Remarks on code availability)

Reviewer #2

(Remarks to the Author)
Zhang et al. present an experimental investigation of mixed-state phases on a quantum computer using Rényi correlators. The results are clearly presented and well explained in the revised manuscript. The revised version is concise, well-motivated, and represents a substantial improvement over the previous submission. At this stage, I have only the following minor comments. I would recommend accepting the manuscript once the authors have addressed these points.

Comments
1. The authors state that the decoding fidelity depends on the error rate. Are there any phenomenological observations from numerics—such as a threshold behaviour or scaling—with respect to the number of qubits and the per-gate error rates? This would be valuable in assessing experimental feasibility for large systems.
2. Regarding Jackkniging error bars, the authors' explanation resolves my concern in the first version. I had initially missed that only a single measurement shot is recorded per random unitary. I am satisfied with their response.
3. The authors mention that IonQ has achieved 99.99% fidelity. It would be helpful to include a citation. I assume this refers to Hughes et al., arXiv:2510.17286 (2025). For clarity, please note that this fidelity reported for a two-ion system.
4. It would improve readability if the first 45 references were distributed more evenly throughout the introduction, as the current clustering makes it difficult to follow which works are relevant to which statements.
5. In the second paragraph, please use n≥2 instead of n>1for consistency.
6. Figure 1, in the caption, authors state 20 physical qubits. Could the authors clarify how these are divided between logical qubits and ancillary qubits?
7. In the definition of the Hamiltonian, the signs of the XX and Z terms are negative. Could the authors explain how these negative signs was implemented in the experimental setup?
8. The authors use a U3 design. Please comment on why this was chosen instead of Haar-random unitaries.
9. If the variational optimization was performed classically using pre-computed parameters, please state this explicitly.
10. The manuscript states that the decoding circuit was optimized locally on a classical computer. Does this imply that all experimental inputs were included in the optimization? If so, please mention this either in the main text or in the Supplementary Material.
11. It is still unclear whether the dephasing model used was local or global. A brief clarification would be helpful.
12. In the Supplementary Material, the stated 9% infidelity for correlators is not fully explained. Based on the provided error rates, I do not immediately see how this value is obtained. A brief explanation would suffice.
13. The caption of figure 13 appears to be missing an "L". I believe the authors meant L=4,8.

(Remarks on code availability)

Reviewer #3

(Remarks to the Author)
I appreciate the effort the authors put into revising their manuscript in response to the referees' comments. Now the motivation of the proposed approach is clearer, and, in particular, the quality of the experimental data has improved significantly. All these modifications improved the work overall, and I am now open to supporting the revised version for publication, provided that the authors address the points below.

1. It is still not clear whether the use of variational decoding for distinguishing mixed-state phases can be extended more broadly to classes of mixed-state phases beyond critical ground states under decoherence, the specific case tackled in this paper. This limits the relevance of the proposed method as a general diagnostic tool and makes its broader impact on the study of mixed-state phases somewhat uncertain. A more in-depth discussion in the outlook about possible generalizations would further strengthen the paper.

2. At the beginning of the last paragraph on page 3, the authors wrote, "In terms of the amplitude of $C_{XX}^{(2)}(l)$, we observe a significant increase under X dephasing ...". However, based on both Fig. 2 and Eq. (2), should we not expect it to saturate or to slightly decrease due to finite-size effects, as the authors point out in the caption? Maybe the authors mean X increases and Z decreases with respect to the identity channel? A clarification is needed.

3. Regarding the description of the behavior of F_e with respect to pL^{1/2} for Z dephasing, the term ``non-monotonic" may not be precise, as it appears to vary rather smoothly. I think the authors should clarify their meaning.

(Remarks on code availability)

Reviewer #4

(Remarks to the Author)
I co-reviewed this manuscript with one of the reviewers who provided the listed reports. This is part of the Nature Communications initiative to facilitate training in peer review and to provide appropriate recognition for Early Career Researchers who co-review manuscripts.

(Remarks on code availability)

Version 2:

Reviewer comments:

Reviewer #2

(Remarks to the Author)
All previous queries have been adequately addressed. I have no further comments and therefore recommend the manuscript for publication in Nature Communications.

(Remarks on code availability)

Reviewer #3

(Remarks to the Author)
We have carefully examined the revised manuscript, the supplementary material, and the authors’ responses to all reviewers’ comments. Considering the authors’ other responses and the additional material included in the revision, such as the application of variational decoding to symmetry-protected topological phases, we recommend publication in its present form.

Regarding the scalability issue raised by Reviewer 1 regarding the variational approach, we find the authors’ response convincing. That said, readers would benefit from an explicit clarification in the main text that the approach applies only to a local decoder and to correlations that evolve smoothly with the system size.

As a final minor point, we would appreciate knowing the fitted slope for $C_{xx}^{(2)}(l)$ for the identity channel as well, since this would provide a better benchmark of processor performance. In particular, the exact numerical data appear to track the experimental data in both dephasing cases, but not in the identity-channel case in Figure 2(b).

(Remarks on code availability)

Reviewer #4

(Remarks to the Author)
I co-reviewed this manuscript with one of the reviewers who provided the listed reports. This is part of the Nature Communications initiative to facilitate training in peer review and to provide appropriate recognition for Early Career Researchers who co-review manuscripts.

(Remarks on code availability)

Open Access This Peer Review File is licensed under a Creative Commons Attribution 4.0 International License, which permits use, sharing, adaptation, distribution and reproduction in any medium or format, as long as you give appropriate credit to the original author(s) and the source, provide a link to the Creative Commons license, and indicate if changes were made.
In cases where reviewers are anonymous, credit should be given to 'Anonymous Referee' and the source.
The images or other third party material in this Peer Review File are included in the article's Creative Commons license, unless indicated otherwise in a credit line to the material. If material is not included in the article's Creative Commons license and your intended use is not permitted by statutory regulation or exceeds the permitted use, you will need to obtain permission directly from the copyright holder.
To view a copy of this license, visit https://creativecommons.org/licenses/by/4.0/

RESPONSE TO REFEREE

Dear Referees,

First of all, we thank you for carefully reading our manuscript. We are encouraged that the reports describe the work as “very interesting,” “promising,” and “solid.” We are grateful for your many constructive comments and have taken them to heart. Below we address the three of your main concerns: (1) the motivation for Rényi correlators, (2) the novelty and scalability of the variational decoding, and (3) the quality and clarity of the experimental results.

(1) *Motivation for Rényi correlators.* We now make explicit that our use of the doubled Hilbert space (replica/thermofield double framework) for mixed-state phases is standard, so defining two-point correlators in this setting is natural and theoretically meaningful. Our revised text explains this motivation up front and restricts claims to what our system sizes support. Importantly, our results offer two novelties: (i) mixed-state phases can be distinguished without resorting to negativity or higher-order ($n \geq 3$) Rényi measures: second-order Rényi correlators already separate the regimes; and (ii) the observed behavior disagrees with a commonly believed CFT expectation for this setting. We have rewritten the relevant sections to reflect these points clearly and conservatively.

(2) *Novelty and scalability of variational decoding.* We realized our previous presentation under-emphasized the scaling evidence. We have therefore added new numerical experiments up to $L = 24$ focusing on decoder performance and sample complexity. These results clarify two contributions: (i) gapless mixed-state phases can be separated at shallow circuit depth with polynomial measurement cost; and (ii) the approach extends to physically motivated AQEC codes whose decoders are not known a priori. Due to the sample efficiency, low-gate-requirement properties of variational decoding method, it stands strong as a candidate for studying mixed state phases in near term quantum computers. Previously, there is no proposal for probing mixed state phases efficiently on fault-tolerant quantum computers: our extended numerical study suggests a strong, affirmative answer.

(3) *Experimental quality and interpretability.* Thanks to recent hardware improvements on Quantinuum H1, the separation between X- and Z-dephasing channels is now clearly observed, addressing the earlier blur highlighted by Reviewer 3. We have also made the presentation substantially clearer and summarized an error budget in SM. As per referees' suggestion, we have added a panel in Fig. 1 to illustrate the geometry of the hardware used in this work.

Beyond these major changes, we tightened the language throughout, added missing experimental details and error analysis, and reorganized the Supplementary Material with clear sectioning and cross-references from the main text. We believe that the changes made have improved our paper and that the current manuscript will be deemed suitable for publication in *Nature Communications*.

Sincerely yours,

Yuxuan Zhang, Timothy H. Hsieh, Yong Baek Kim, and Yijian Zou

Reviewer #1 (Remarks to the Author):

The submitted manuscript studies the effects of dephasing on the structure of a quantum state prepared in a quantum device. The authors focus on so-called Renyi correlators, and on the entanglement fidelity. These objects have been studied in many recent theoretical works aiming to classify quantum states generated by local decoherence. The experimental results (in Figs. 2 and 5) are the observations that (i) after X errors, the Renyi correlator decays more slowly with separation than in the initial state, (ii) after Z errors, it decays more quickly, (iii) the entanglement fidelity following Z errors is higher than after X errors.

Reply: We thank the referee for carefully reading our manuscript and summarizing our results. However, we would like to emphasize that the statement "entanglement fidelity following Z errors is higher than after X errors" is not very precise. Rather, asymptotically, the X-errors are uncorrectable for the CFT code when the system is subject to total number of errors beyond $\sim L^{1/4}$; but $O(L)$ Z-errors are still correctable. The entanglement fidelity at finite sizes reflect the asymptotic behavior. Adding in more physical qubits increases the entanglement fidelity for Z dephasing but decreases for the X dephasing in the presence of $\sim L^{1/2}$ errors.

The entanglement fidelity has a clear quantum information theoretic meaning, and the method for measuring it is novel. The Renyi correlator does not have such a clear meaning, and can only be extracted using exponential resources (as discussed by the authors). I appreciate that the Renyi correlator is of interest for the community of theorists actively working on classifying mixed state phases but here its investigation is not very well motivated. Here it comes across as one of (many) objects that can be constructed from shadows.

Reply: The Renyi-2 correlator is the correlation function of the vectorized state $|\rho\rangle\rangle$, i.e., the state in the doubled Hilbert space, which has been used by condensed matter theorists to classify mixed-state phases. To this end, the Renyi-2 correlator we propose here is the lowest-order correlator that can distinguish the two mixed-state phase in critical systems, while previously only Renyi-3 negativity is known. Our proposed Renyi-2 correlator has smaller shadow norm, thus requiring less measurement budgets than the previously known quantity.

The experiment studies systems of up to eight qubits prepared in an approximation to the critical Ising ground state. However, the initial quantum state is not characterized. For example, in Fig. 2(b) no experimental data is shown for the case without dephasing, and no information is provided on how the state is prepared (aside from the fact that a variational quantum circuit is used). The authors' interpretation of their result hinges on this state having correlations resembling those of the true critical Ising ground state, so it seems necessary to at least show conventional correlation functions compared with the corresponding finite-size numerical results.

Reply: We thank the referee for bringing up the point. In response to this, we have included experimental data for the identity channel, hich is within $\sim 10\%$ error from numerical simulation (similar to other channels). Further, we have included more technical details in SM on the initial state preparation.

Should we believe that Ising CFT is a relevant framework for describing the effects of decoherence on six (or even eight) qubits?

Reply: Yes, the Ising model is known for its small finite-size corrections and the CFT behavior already emerges at small system sizes. For example, the one can numerically extract the scaling

dimensions of the spin operator σ and energy operator ε from the energy spectrum. At $L = 8$ sites, the numerical value is $\Delta_\sigma = 0.130$ and $\Delta_\varepsilon = 1.03$, which agrees in 4% relative error from the CFT predictions (0.125 and 1, respectively). This finite-size correction is further suppressed as $1/L^2$ as the size increases. Therefore, we expect that the Ising CFT gives a correct prediction even at small sizes. Indeed, in Fig. 2(b), the $L = 6$ result already shows agreement with the CFT prediction, with X dephasing enhancing the Renyi correlator to a slower decay and Z dephasing deceasing the Renyi correlator to a faster power-law decay, although the exponents are a bit off the exact value due to finite-size corrections. In the new decoding part, we also simulate the variational decoding approach up to $L = 24$ spins, where the finite-size corrections from CFT become negligible.

In the abstract, the authors write that they use shadow tomography to observe a Renyi correlator in two distinct phases, one having power-law correlations and the other having long-range correlations. This is pretty unclear: numerical calculations in large systems show a significant difference in correlations, but it is not possible to say whether the experiment detects long-range correlations in one case and power-law correlations in the other: There are only three experimental data points for each data set in Fig. 2. It would be fair to say that the experiment detects e.g. 'a larger Renyi correlator' in one case.

Reply: We thank the referee for the suggestion. We have changed to the sentence in the abstract to "one with enhanced correlator and slower decay and the other with decreased correlator and faster decay. "

In Fig. 1 it would be helpful if

1) *The geometry of the experimental system is displayed. Is this a system with open boundary conditions, with correlations between two sites away from the ends of the chain, or between one end and a site in the middle?*
2) *There is a standard scale in Fig. 2(b), in units of sites. At present it is not clear what the data shows.*
3) *Finite size experimental results are compared with numerical predictions (shown as points) for the finite size chain.At present, the information provided about Fig. 2(b) is insufficient to understand what is being displayed.*

Reply: We thank the referee for the suggestion. We have revised the manuscript to reflect these points throughout the main text and appendix. Specifically:

1) The experimental system has 20 qubits arranged on a 1d lattice, as we illustrate in the new Fig. 1(c). Despite the 1d architecture, it allows physically moving the ion-traps, effectively enabling an all-to-all connectivity. In the experiment we are using the neighboring qubits towards the one end of the chain and long-range correlations are created by the sequential circuit.
2) In Fig. 2(b), we show the Renyi-2 XX correlator $C^{(2)}_{XX}(l)$ with $l = 1, 2, 3$ in a system with $L = 6$ spins. The horizontal axis is $\big(L/\pi\big)\sin(\pi l/L)$, which is the chord distance between two sites on a ring. This is a standard choice for conformal correlators on ring, justified through the following conformal mapping. The conformal mapping from a plane to a cylinder $z = e^{2\pi w/L}$ rescales distances by $|dz/dw| = 2\pi/L$. On an equal-time slice of the cylinder of circumference L, two points separated by arc length l on the cylinder map to a chord distance on the plane $|z_{12}| \sim \sin\big(\frac{\pi l}{L}\big)$, with the L/π chosen as "rescaling" factor.

We have also taken the log-log scale such that the slope of the line reflects the critical exponents.

3) We have included a better explanation of Fig 2b in the caption. The line represents exact simulation result and the dots represent experimental data.

As a general point, the lines between interpretation, theory, and experimental results are not particularly clear. To improve the clarity of the paper, it seems that essential that the captions of Figs. 3 and 4 state explicitly that these are numerical rather than experimental results.

Reply: We thank the referee for their suggestion. We have clarified the distinction between numerical and experimental results more clearly. We explicitly add "from numerical simulations" to the captions of Fig. 3 and the updated version of Fig. 4, to distinguish it from "experimental results" in the caption of Fig. 5.

The claim in the introduction that the initial state can be interpreted as a quantum error correcting code has the potential to be extremely confusing to readers who are not in the theoretical community actively working on these problems. In particular, the authors write 'Traditional QEC codes, typically based on the stabilizer formalism and exemplified by topological codes, require implementation in at least two spatial dimensions. On the other hand, recent theoretical studies have shown that many physical states, even in one dimension, can be used as QEC codes'. This sentence creates the impression that the states studied here can be viewed as useful QEC codes. The reality is that QEC is a useful framework that can be applied to the study of mixed state phases. It seems important to maintain a sharp distinction between actual QEC, on the one hand, and CMT interpretations through the lens of QEC, on the other.

Reply: We thank the referee for noticing the possible confusion. We have changed the sentence in the introduction to "One particularly interesting insight of the mixed-state phases comes from quantum error correction codes (QECCs), as the mixed-state phase transitions coincide with decodability transitions in particular QECCs known as CFT codes". We no longer hint at using CFT codes as a practical protocol but stress more on the conceptual bridge between mixed-state phases and QEC.

The line 'they are typically characterized by nonlinear observables and information-theoretic quantities' is likely to be confusing to readers outside outside of the theoretical community working on these problems. 'Nonlinear' in what?

Reply: These quantities are nonlinear in the density matrix. We changed the sentence to "Despite the theoretical formulation, it has been challenging to observe mixed-state phases and their code properties experimentally because they are typically characterized by quantities that are nonlinear in the density matrix, such as the coherent information ."

There is a conjecture in the discussion that references numerical calculations that are not included in the manuscript: 'Based on our numerical simulations on small sizes, we conjecture that optimally decoding a correctable dephasing error at a finite rate requires the circuit layer scaling as tau = O(L)... '. This leads to the possibility of detecting difference mixed state phases in polynomial cost. In connection with this, it would be very useful if the numerical results which are the basis for this conjecture are included.

Reply: Previously, our conjecture about optimal decoding depth comes from numerical simulation of small sizes $L = 3, 4, 5$, where the optimal depth is reached at $\tau = 5, 7, 13$, respectively. However, we feel the numerical results were limited by their system sizes and thus we have moved

the discussion to SM. Instead, we provide new evidence that a suboptimal decoder at shallow depth is sufficient to **distinguish the two phases**. In particular, we show in the new Fig. 4 that a shallow circuit with depth $d = 3$ is sufficient to distinguish the two mixed-state phases up to $L = 24$ spins through a tensor network simulation. For fixed $pL^{1/2}$, F_e is monotonically decreasing with L for the X dephasing but increase with L for the Z dephasing when $pL^{1/2} \geq 1$. The latter non-monotonic behavior of F_e comes from the competition between limited decoding depth and the increase of optimal decoding fidelity with L. This reflects the difference of decodablility in the thermodynamic limit, in agreement with the CFT prediction. We further verify that a circuit at $d = 2$ is only sufficient to distinguish the two phases up to $L = 8$. While we cannot conclude the asymptotic scaling of the decoding circuit depth d with L, we see that a shallow circuit is already sufficient for moderate sizes for NISQ platforms.

In connection with this: Do the experimental decoding results in Fig. 5 use decoders constructed from numerics? If so, does this scheme not require either full state tomography at the start, or otherwise the preparation of a quantum state that is (in principle) known exactly?

Reply: The experimental decoder is constructed from a numerical optimization. In the numerical study, we exactly know the prepared state, which is the critical Ising ground state under decoherence. Finding the variational decoding circuit on a quantum computer when the mixed state is unknown does not require full state tomography. Instead, the optimization can be done fully on the quantum computer. The entanglement fidelity is the probability of the all-0 string in the output. Optimizing the fidelity can be achieved with a standard variational algorithm.

The authors write that, with a decoder of unit depth, the experimental entanglement fidelity is already different for X and for Z errors. Is it reasonable to expect that a local circuit would be sufficient here, given that deep circuits were required for numerics?

Reply: This is a very good question. In the revised manuscript, we've conducted extensive numerics to address this concern. Comparing Fig. 13 (a) in the SM and Fig. 4 in the main text, it can be seen that at decoding depth 1, the variational decoder successfully differentiates the two channels at $L = 4, 8$ but struggles for larger system sizes. At larger decoding circuit depth, such as depth 3, our new numerical results show that it can distinguish the two phases up to $L = 24$. We take this as a strong evidence that a relatively shallow circuit would be sufficient.

The method for measuring F_e (used in Fig. 5) is currently illustrated in Fig. 1 but is not described in the main text. In practice the circuit used for initial state preparation, as well as its inverse, are subject to errors. When these errors are taken into account, is the entanglement fidelity that is eventually measured still a lower bound on the true entanglement fidelity?

Reply: We thank the referee for observing this nuanced difference. F_e is measured through the frequency of all 0 output in the final measurement. In the most general case, the presence of additional quantum channel may bias the output toward the all-0 string and thus increase F_e. Nevertheless, in a real quantum device, it is usually reasonable to consider the effect of physical noise, as a whole, as a circuit-agnostic depolarizing channel, in which case the measured entanglement fidelity still a lower bound on the true quantity.

In summary, the experimental results in this paper would benefit from more explicit explanations. Since the question of whether mixed state phases can be observed is essentially open, with papers even claiming that they cannot be observed directly in data (see arXiv:2504.12233), a proof of concept

for such an experiment should provide a detailed analysis of the resource requirements. While the theoretical and numerical results are very interesting, these sit apart from the experimental results. That said, my overall view is that neither the theoretical nor experimental results (taken on their own) merit publication in Nature Communications.

We thank the referee for the thoughtful assessment and for pointing out `arXiv:2504.12233`. While it has been an open debate that whether mixed-state phases can be effectively observed, our latest numerical result serves as a solid evidence suggesting an affirmative answer. We would like to point out an important distinction here: The referred work studies a particular notion of mixed-state phase, i.e., a "pseudo-strong-to-weak SSB", and its unlearnability result is proven for a carefully constructed family of instances. This does not establish that operationally meaningful mixed-state phases are unobservable on physically relevant states. Indeed, even for pure states one can engineer hard observables, e.g., unlearnability of pure state phases based on low-depth pseudo-random unitary `arxiv:2407.07754`, that are not efficiently learnable in the worst case, but it would be unjustified to conclude that all pure-state phases are unobservable.

In our work, our target phases belong to a particular class of mixed-state phases, i.e., critical ground states under decoherence. The variational decoding algorithm is particularly chosen to distinguish the critical mixed state phases. With our improved numerics and experiments, we show that, despite noise, our protocol remains a credible near-term route to observing mixed-state phases. We also include concrete resource estimates to make this feasibility transparent. Therefore, we believe that our work opens a path on observing other physically relevant mixed-state phases on quantum computers despite the hardness proof of generic cases.

Reviewer #2 (Remarks to the Author):

In this manuscript, Zhang et al. experimentally studied mixed-state phases via Rényi correlators. The study was carried out on Quantinuum's H1 processor. The authors claim to demonstrate characterization methods for mixed-state phases. Specifically, under controlled decoherence channels, the decoding capability of error-correcting codes using variational circuits was tested. The study is presented in two parts. First, Rényi correlators via classical shadows were used to distinguish mixed-state phases. The results are corroborated with conformal field theory (CFT) calculations. These findings were complemented by entanglement fidelity diagnostics. Second, the authors tested the decoding fidelity associated with error-correcting codes. I find the results overall satisfactory. I would like to emphasize that these diagnostic tools are essential for quantum simulation platforms, where distinct features of mixed-state phases can become important in studying universality classes of interactions.

Reply: We thank the referee for their favorable assessment.

Detailed Comments:

1) *I wonder if these kinds of tests are more appropriate for larger system sizes and more complex quantum states, where full state tomography becomes impractical. In this study, the system size is relatively small—up to 8 qubits. While I still find the results promising, it is unclear to me what the key novelty of the presented case study is. A clearer articulation of the conceptual or methodological advancement, beyond demonstrating feasibility on a small system, would strengthen the manuscript.*

Reply: We have now extended simulation of the decoding circuit up to $L = 24$ qubits, a significant improvement from the previous simulations. Although the experiment still focuses on $L = 8$ case, we have also estimated that the $L = 24$ experiment would be feasible given that the two-qubit gate fidelity reaches 99.99%, a goal in near feature quantum devices. Our key novelty is the variational construction of decoding circuits, which can be used to distinguish mixed-state phases when the decoder is known a priori (e.g., not a stabilizer code). The Renyi correlator also displays a surprising exponent in its subleading term, which goes beyond current understanding of boundary CFT. This could be of separate interest to field theory experts.

2) *The experimental error bars in Figures 2 and 3 appear to be unusually small. Could the authors clarify the measurement budget? Specifically, how many random unitaries were implemented, and how many shots per random unitary were taken? Additionally, what assumptions underlie the reported error bars? They mentioned Jackknifing, on what this Jacknifing was performed. Understanding this would help evaluate the statistical reliability of the results.*

Reply: We have included a more careful budget and error analysis in the revised manuscript. Fig.3 is a theory plot from numerical simulation. For Fig.2b (Renyi correlator) For each shot, we draw a random unitary on all sites and measure in the Z basis. In total, we have used 40000 shots for estimation of the Renyi correlators. The Jacknifing is a standard statistical tool for estimating the error bars in classical shadow reconstructions (see also [Phys. Rev. Lett. 125, 200501]. The variance is estimated in the following way: 1) For the 40,000 shots we collect, we create a subsample by omitting one shot and re-compute estimated Renyi-correlator on the subsample. 2) The first step is performed 40,000 times, each time omitting a different shot. We thus collect 40,000 different estimates of the Renyi correlators. 3) We estimate the bias and calculate their variance of these 40,000 estimates.

3) Did the authors characterize the intrinsic (physical) noise affecting their observations, beyond the engineered noise channels they introduced? For example, in the presence of engineered X-type noise, are there other background noise sources that might contribute to discrepancies between theory and experiment? The manuscript briefly mentions gate infidelities—could these also influence the observed behavior? Furthermore, the authors introduce dephasing channels using additional random operations. Can these be reasonably modeled as white noise, which is often the assumption in theoretical calculations? It would be helpful to include a more detailed discussion of these aspects in the manuscript.

Reply: Yes! Upon the referee's suggestion, we have now included more details in the error analysis in the SM, section D. In short, we find that there are two types of noises that dominate in the physical realization: The first is a two qubit gate error, usually characterized as a depolarization channel (white noise); The second is a measurement (SPAM) error, usually characterized as a bit-flip error.

4) *The manuscript should mention the system size clearly and early on. For instance, beginning with the third paragraph of the Introduction, a sentence such as: "In this work, we... on a trapped-ion quantum processor with N= 5-8 qubits" would help contextualize the scale of the experiment right away.*

Reply: We have added the sentence that "We demonstrate our proposal on a trapped-ion quantum processor using $4-8$ spins." In the meantime, we want to emphasize that our numerical simulation shows that the $L = 24$ experiments could be achieved with depth 3 circuits when the two-qubit gate fidelity reaches 99.99%, a value that just have been reached on small system size demonstration by IonQ.

5) *The Supplementary Material (SM) is difficult to follow without any sectional structure. It would be helpful if the SM were divided into clearly labeled subsections. Furthermore, references to the SM within the main text should include specific section numbers to improve navigation.*

   Reply: We thank the referee for their point and have labelled the SM as the referee suggested. A table of content is included.

6) *I was confused by the simulation results shown for L=64, especially considering the largest experimental system size used is only N=8. If conformal field theory (CFT) validation requires large system sizes, this should be explicitly stated and discussed in the manuscript to avoid ambiguity.*

   Reply: The CFT validation does not require a large size. In fact, the Ising model is known for its small finite-size corrections and the CFT behavior already emerges at small system sizes. For example, the one can numerically extract the scaling dimensions of the spin operator σ and energy operator ε from the energy spectrum. At $L = 8$ sites, the numerical value is $\Delta_\sigma = 0.130$ and $\Delta_\varepsilon = 1.03$, which agrees in 4% relative error from the CFT predictions (0.125 and 1, respectively). This finite-size correction is further suppressed as $1/L^2$ as the size increases. Therefore, we expect that the Ising CFT gives a correct prediction even at small sizes. Indeed, in Fig. 2(b), the $L = 6$ result already shows agreement with the CFT prediction, with X dephasing enhancing the Renyi correlator to a slower decay and Z dephasing deceasing the Renyi correlator to a faster power-law decay, although the exponents are a bit off the exact value due to finite-size corrections.

7) *What is the motivation behind choosing the transverse-field Ising model for this study? Would similar behavior be expected for other Hamiltonians, or are the observed features specific to the ground state of the transverse-field Ising model? While I understand that ground state preparation is perhaps easy for this model, a brief justification would help clarify its role in the context of the study.*

   Reply: The critical TFIM is chosen because it has very small finite-size corrections, with the CFT behavior emerging at small sizes such as $L = 8$. Also, the ground state is sufficiently low entangled, which can be prepared much efficiently. As a consequence, we only use a depth-1 sequential circuit to reach ground state fidelity 99% for $L = 8$. For generic CFTs, it is predicted theoretically that if the fusion algebra of the jump operators have minimal scaling dimension $\Delta > 1/2$, then the CFT code has a finite decoding threshold. On the other hand side, if $\Delta < 1/2$, then it does not have a finite decoding threshold. The prediction only depends on the CFT, but not the lattice realization. Therefore, for any realization of the Ising CFT on the lattice, a strongly symmetric dephasing noise always has finite threshold, whereas any dephasing channel with jump operators proportional to

the Z_2 odd spin operator does not have a finite threshold.

8) *The authors study two noise channels separately. Could there be any nontrivial interplay if both noise channels act simultaneously? Would the current estimators be capable of distinguishing the corresponding mixed-state phases under such combined noise? Including a brief discussion or comment on this point will improve understanding the role of noises and their combined effects.*

   Reply: We thank the referee for their suggestion. In fact, any noise combined with X noise does not have a finite decoding threshold because the X noise does not. For example, the combined Z and X noises show the same decoding behavior as the X noise.

I find the overall results promising and potentially impactful, opening the door to several interesting research directions, as already pointed out in the outlook of the manuscript. In particular, bridging concepts from quantum error correction, quantum simulations, and conformal field theory offers a compelling and rich framework for future exploration. However, the presentation of the manuscript could be significantly improved for better readability. Key technical details—such as the measurement budget, error analysis, and specific information about the quantum system used—are insufficiently discussed. With a thorough revision that addresses these points, I believe the manuscript could be suitable for publication.

Reply: We thank the referee for the favorable assessment and feedback. In the latest version we have improved the quality of presentation, discussing all the points mentioned by the referee, including measurement budget, error analysis, and specific information about the quantum system used.

Reviewer #3 (Remarks to the Author):

The manuscript by Zhang et al. reports on the experimental realization of mixed-state phases on the Quantinuum H1 quantum computer and the characterization of a corresponding CFT-based QEC code with the implementation of a decoder for small system sizes. More specifically, they prepare small (L = 4–8) ground states of the critical transverse-field Ising chain, and apply engineered X or Z dephasing, and then probe the resulting mixed-state phases with two complementary diagnostics: (i) Second-order Rényi two-point correlators, reconstructed with classical-shadow tomography and (ii) a ladder-ansatz variational decoder that maximises entanglement fidelity Fe between the logical subspace and a reference qubit. Their theory shows that even with a single decoder layer, the hardware data already separates the two phases: Fe rises with L under Z noise but falls with L under X noise as predicted by their previous theory work [PRL 133 210601 (2024)].

While this work is definitely interesting and worthy of publication, I do not think it hits the bar of novelty required for publication in Nature Communications for the following reasons:

1. *While the manuscript convincingly demonstrates the first experimental probes of CFT-based mixed-state phases and implements the first working decoder for such a code, all the theory advances were already established in the recent paper PRL 133 210601 (2024) coauthored by two of the authors of this work. The novelties of this work are the implementation of the decoder and the numerical evidence, through a greedy algorithm, that finding the decoder variationally can be done in O(1) steps for small system sizes. My opinion is that this is a follow-up to their previous work, which doesn't add a significant degree of novelty to warrant publication in Nat. Comm., also because the experimental evidence, even if gathered for more system sizes, is not very convincing (see below points 3. and 4.).*

Reply: We are delighted to see the comment that "the manuscript convincingly demonstrates the first experimental probes of CFT-based mixed-state phases and implements the first working decoder for such a code". With new results in the revised manuscript, we believe that our work now goes significantly beyond our previous PRL work. As we demonstrate numerically in the new Fig. 4, a shallow decoder at depth 3 is sufficient to distinguish the two mixed-state phases up to $L = 24$ spins. The existence of such a shallow decoder indicates that the mixed-state phases can be observed on NISQ devices. While the experiment is for small system sizes up to $L = 8$, we estimate that the $L = 24$ experiment could be done if the two-qubit gate fidelity reaches 99.99% in the near future. Given that whether the mixed-state phase can be efficiently observed is still under debate, our work offers convincingly positive evidence on observing nontrivial critical mixed-state phases on NISQ devices. We have also improved our experimental results due to recent hardware improvement of Quantinuum H1, see reply to points below.

2. *The evidence gathered at small system sizes suggests that decoding is efficient, but only if the decoder does not have an exponential number of gates, and it is not clear when this condition occurs. The conjecture in the outlook that the number of layers in the decoder scales linearly with the system size is not well motivated. Also, it is not clear what they mean by "it grows much more slowly" in the uncorrelated dephasing phase: logarithmically? O(1)? Since this is one of the main takeaways of the paper, I think the authors show limited evidence for it.*

   Reply: We have removed the statement about the optimal decoding depth, but provided new evidence that a suboptimial decoder at small depth $d = 3$ can be used to distinguish the two mixed-state phases up to $L = 24$. We also show that $d = 1$ and $d = 2$ circuit can only distinguish the two phases up to $L = 8$. Although the asymptotic growth of d needed to distinguish the two phases with respect to L is not clear at the moment, we believe that the growth is likely slower than polynomial given the three data points.

3. *About the experimental evidence, in Figure 4, it is not clear that the claim that the decoder performance for X-noise nearly saturates at $\tau = 5$ for all three sizes is justified, especially for the L=5 case. Probably it is more like tau=15 in the L=5 case.*

   Reply: We agree that the saturation of decoding fidelity at small depth is less well supported and We have removed the statement. In the new numerical study, we instead focus on distinguishing the two mixed-state phases rather than achieving optimal decoding. We show that even at shallow decoder depth $\tau = 3$ the X and Z channels can be distinguished up to size $L = 24$. In the new SM we also estimate that such distinction can be observed experimentally in the next few years based on an error analysis.

4. *In Figure 5, the improvement from L=4 to L=8 at $pL^{1/2} > 1$ can be really seen only in the theory curves. If one looks at the error bars, all those data points seem statistically compatible, so it is a bit of a stretch to say that the authors verified experimentally that there is a region where the L=8 code outperforms the L=4 code. If there were no theoretical curve, one would not even have guessed that there is a crossing point around $pL^{1/2}$=1. So the claim that the authors experimentally verify this feature is a bit overstated.*

Reply: In the revised manuscript, we make it clear that the crossing for the Z dephasing is the key to distinguish it from the X dephasing. For Z dephasing, the increase of optimal decoding fidelity competes with finite decoding depth, leading to non-monotonic behavior of the actual decoding fidelity at finite depth d, whereas for X dephasing, there is no such competition as the optimal decoding fidelity also decreases with L. We have now observed the crossing up to $L = 24$ for circuit depth $d = 3$, confirming the expectation for much larger system sizes. Additionally, we have the improved quality of our experimental data, which clearly indicates the observation of a crossing.

5. *The present code is not universal as it does not allow for corrections for X depolarizing noise (as already pointed out in the 2024 PRL). Therefore, from the application point of view is a bit limited.*

   Reply: The work is not intended to be used as a practical QEC scheme, but rather a way to distinguish mixed-state phases on NISQ based on their QEC properties. Thus, the existence both a non-decodable phase and a decodable phase is suited for demonstration of their distinctions. One could instead consider a CFT code where all dephasing is decodable (i.e., all scaling dimensions greater than 1/2), which has better QEC properties but only one mixed-state phase at small noise rate.

Overall, this work seems a solid follow-up of the 2024 PRL, where small-scale experimental implementation has been realized (with some limitations) and decoding with a variational approach has been fleshed out. However, I don't think this is enough to warrant publication in Nature Communications.

Reply: We thank the referee for their perspective, but we respectfully disagree that the present manuscript is merely an incremental follow-up of PRL (2024). Beyond recapitulating prior theory, we (i) introduce and experimentally access a Rényi-2 correlator that cleanly separates the two mixed-state phases—something not demonstrated before; (ii) implement the first working decoder on hardware and provide new scaling evidence that a shallow, depth-3 variational decoder distinguishes the phases up to $L = 24$ with tensor-network numerics, identifying a concrete near-term route to mixed-state phases; and (iii) substantially upgrade the experiment on Quantinuum H1, where the X/Z separation is now clearly resolved, and we add a detailed feasibility/resource analysis for future works. Taken together, these additions go well beyond the 2024 PRL: they close the theory–experiment loop for mixed-state phases with a practically learnable protocol and provide new evidence on scalability. We believe that our approach offers a new path towards observation of other mixed state phases in the future. Therefore, we believe meets the bar for *Nature Communications.*

Other Clarifications:

1. *How would the results in Figures 2 and 3 change when $p > 0.3$?*

   Reply: The numerical simulation of Renyi correlators under different dephasing channels and different p's is presented in SM Section A.2 and A.3. For the X dephasing, the Renyi-2 XX correlator is shown in Fig. 7 left panel, where all p's give rise to saturation to a constant at large distances. The constant is non-universal and increases with p, but the convergence to the constant follows the same power law scaling (Fig. 7, right panel). For the Z dephasing, a larger p gives a larger exponent η_2^Z, which continuously changes with p (Fig. 9). The qualitative behavior is the same across all p's: for X dephasing the correlator

saturates to a constant. For Z dephasing the correlator has a faster power-law decay. Fig. 3 already extends to $p = 0.5$.

2. *It is not clear in the first sentence of the last paragraph of the first column on page 5, what is "d=1". Since D=2 throughout the paper, maybe it refers to dimensionality. Can the authors clarify?*

   Reply: We use d to refer to the circuit depth of the decoding circuit. D denotes the code subspace dimension, which equals 2 in our setting.

Reviewer #4 (Remarks to the Author):

I co-reviewed this manuscript with one of the reviewers who provided the listed reports. This is part of the Nature Communications initiative to facilitate training in peer review and to provide appropriate recognition for Early Career Researchers who co-review manuscripts.

RESPONSE TO REVIEWER COMMENTS

REVIEWER COMMENTS

Reviewer #1 (Remarks to the Author): Thank for sending this article, which has improved since the initial submission. However, I have technical concerns about the analysis of the experimental data, and about the claimed efficiency of the variational decoding method. Overall, while the theoretical results are interesting, I still do not find that the experimental results or their analysis merit publication in Nature Communications.

1. In Fig. 2 the authors fit a power law to an experimental dataset consisting of just three points, and where neither the horizontal or vertical axis values change by anywhere close to one order of magnitude. It is difficult to take the results of this fit, and the corresponding exponents, seriously. This exponent is also quoted without an error bar.

   We thank the referee for their comments and have rephrased our claim in the abstract as "we employ shadow tomography to observe a newly proposed Renyi correlator in two distinct phases obtained by applying different channels, observing one with faster decay and the other with slower decay.". For the power law scaling, we also added an estimation of the error bar of the fitted exponent. While the fitting is performed on three points, and the error bar is large, the fitted exponents 0.31(7) (Z dephasing) and 0.07(8) (X dephasing) are consistent with the field-theoretic prediction that slope for Z dephasing is greater than 0.25 (the slope for the identity channel) and the slope for X dephasing is 0 as $L \to \infty$.

2. In Fig. 3 the authors compare F_e to the channel distance d_ρ, but how is d_ρ determined? Are the dotted and dashed lines numerical calculations, or is d_ρ determined using full tomography of the experimental state? If numerics, is it reasonable or even meaningful for the experimental result for F_e saturate the bound (this is mentioned below Eq. 9)? There is room for improvement in the presentation of this part of the manuscript.

   We thank the referee for raising this concern. The whole figure comes from numerical calculation, as indicated in title of Fig. 3, "Optimal entanglement fidelity from numerical simulations." The upper and lower bounds are computed from d_ρ, which is also computed numerically. We have further emphasized this in the main text.

3. In the final paragraph before the discussion, the authors write 'Compared with Renyi observales, which requires exp(O(L)) samples for shadow tomography [79], the sampling complexity for variational decoding is O(1).' I do not understand how this can be the case in general. The sample complexity is only O(1) if the optimal decoder is known to extremely high accuracy. However, if the decoder differs from the optimal one by just a near-identity single-qubit unitary on each site, then estimating the fidelity in experiment requires exponential time. The situation relevant to a typical experiment is where one starts from not knowing the decoder and uses the variational method to find it. But, to optimize the fidelity using a variational method, one needs to evaluate F_e for different variational parameters.

Because F_e is generically exponentially small in L, the sample complexity required to experimentally measure F_e for a particular instance of the decoder is exponentially large in L. This would mean that it takes exponential time to optimize the decoder and then use this method (that is, if optimization is done for the experimentally prepared state). If it is possible to successfully optimize using numerics, then by assumption the state prepared in experiment is easy to store and study on a classical computer. This restricted situation would indeed give O(1) sample complexity in the experiment, but the applicability of the method is then extremely limited. Is an experimental method useful if it only works in settings where one has access to exact numerical simulations?

We thank the referee for raising this point. We agree that the $O(1)$ sample complexity sentence in the original manuscript is intended when the decoder is known. If one attempts to optimize the decoder directly in experiment by repeatedly estimating the entanglement fidelity F_e for random variational parameters, then the referee is correct: the signal can become exponentially small in the linear system size L, and the corresponding sample complexity can become exponentially large. This is a generic difficulty for variational quantum algorithms with global cost functions, closely related to barren-plateau behavior and exponentially weak gradients [1–3].

Nevertheless, we would like to point out that this is not a problem that is unique to variational decoder, but rather to all variational quantum solvers [3]. Crucially, many recent works have been successfully addressed this issue with transfer learning: even when the decoder is not known a priori, our Supplementary Material demonstrates a concrete route to avoid an exponentially vanishing initialization signal [4–6]. We use a transfer-learning strategy in which parameters optimized at smaller L are embedded into the ansatz at larger L, and only the newly added local gates are initialized to the identity. As shown in Appendix, this procedure maintains a non-exponentially-vanishing optimization signal at initialization over the system sizes studied, thereby ruling out the blind 'random start" worst-case scenario in our regime (see SM for details) [4, 6].

More broadly, successfully learning a local decoder does not imply that the full many-body state is classically reconstructible. The intended use case is hybrid classical-quantum: one learns or calibrates a variational local decoder on accessible sizes or simpler models, and then transfers it to larger systems where full classical simulation is no longer available. The utility of the method is precisely that it targets logical information through a sequential-circuit decoder, rather than requiring full state tomography.

We have revised the manuscript to make our point explicit.

References

[1] J. R. McClean, S. Boixo, V. N. Smelyanskiy, R. Babbush, and H. Neven, "Barren plateaus in quantum neural network training landscapes," *Nat. Commun.* **9**, 4812 (2018).

[2] M. Cerezo, A. Sone, T. Volkoff, L. Cincio, and P. J. Coles, "Cost function dependent barren plateaus in shallow parametrized quantum circuits," *Nat. Commun.* **12**, 1791 (2021).

[3] K. Bharti *et al.*, "Noisy intermediate-scale quantum algorithms," *Rev. Mod. Phys.* **94**, 015004 (2022).

[4] H.-Y. Liu, T.-P. Sun, Y.-C. Wu, Y.-J. Han, and G.-P. Guo, "Mitigating barren plateaus with transfer-learning-inspired parameter initializations," *New J. Phys.* **25**, 013039 (2023).

[5] E. Grant, L. Wossnig, M. Ostaszewski, and M. Benedetti, "An initialization strategy for addressing barren plateaus in parametrized quantum circuits," *Quantum* **3**, 214 (2019).

[6] A. Skolik, J. R. McClean, M. Mohseni, P. van der Smagt, and M. Leib, "Layerwise learning for quantum neural networks," *Quantum Mach. Intell.* **3**, 5 (2021).

[7] D. J. Egger, J. Mareček, and S. Woerner, "Warm-starting quantum optimization," *Quantum* **5**, 479 (2021).

Reviewer #2 (Remarks to the Author): Zhang et al. present an experimental investigation of mixed-state phases on a quantum computer using Rényi correlators. The results are clearly presented and well explained in the revised manuscript. The revised version is concise, well-motivated, and represents a substantial improvement over the previous submission. At this stage, I have only the following minor comments. I would recommend accepting the manuscript once the authors have addressed these points.
Comments.

1. The authors state that the decoding fidelity depends on the error rate. Are there any phenomenological observations from numerics—such as a threshold behaviour or scaling—with respect to the number of qubits and the per-gate error rates? This would be valuable in assessing experimental feasibility for large systems.

   We thank the referee for this suggestion. Beyond the finite-size behavior seen in our circuit-level numerics, there is an analytical understanding based on renormalization group flow of the dephasing strength, as proposed in Ref. [51] (Sang, Hsieh, Zou, PRL 2024). The effective (renormalized) noise strength scales as $pL^{1-2\Delta}$, where Δ is the scaling dimension of the jump operator. Thus, entanglement fidelity is expected to have universal finite-size scaling near $p = 0$, i.e.,
   $$F_e(p, L) = f\left(pL^{1-2\Delta}\right).$$
   For the Ising CFT, Z dephasing has $\Delta_Z = 1$ and X dephasing has $\Delta_X = 1/8$, representing irrelevant (correctable) and relevant (uncorrectable) perturbations, respectively. Our finite-size numerics are all consistent with this RG flow picture.

   For correctable noise, there is a finite decoding threshold p_c, and the scaling of F_e near $p \approx p_c$ is expected to have an analogous scaling behavior, $F_e(p, L) = \mathcal{F}\Big((p - p_c)L^{1/\nu}\Big)$.
2. Regarding Jackkniging error bars, the authors' explanation resolves my concern in the first version. I had initially missed that only a single measurement shot is recorded per random unitary. I am satisfied with their response.

   We thank the referee for their comment.

3. The authors mention that IonQ has achieved 99.99% fidelity. It would be helpful to include a citation. I assume this refers to Hughes et al., arXiv:2510.17286 (2025). For clarity, please note that this fidelity reported for a two-ion system.

   We thank the referee for their comment and have included a citation in the manuscript.

4. It would improve readability if the first 45 references were distributed more evenly throughout the introduction, as the current clustering makes it difficult to follow which works are relevant to which statements.

   The introduction has now been modified.

5. In the second paragraph, please use $n \geq 2$ instead of $n > 1$ for consistency.

   We thank the referee for their comment and have updated the manuscript per suggestion.

6. Figure 1, in the caption, authors state 20 physical qubits. Could the authors clarify how these are divided between logical qubits and ancillary qubits?
   The first qubit is reference qubit (that is. logical qubit) and the rest are ordered as physical 1, ancilla 1, physical 2, ancilla 2....

7. In the definition of the Hamiltonian, the signs of the XX and Z terms are negative. Could the authors explain how these negative signs was implemented in the experimental setup?
   We directly prepare the ground state of H (which has a minus sign) using a variational circuit. The circuit is optimized fully classically and implemented on the quantum computer, but would be eventually optimized using hybrid quantum-classical optimization scheme. Therefore, the minus sign only appears in the classical part of the optimization and does not cost additional overhead.

8. The authors use a U3 design. Please comment on why this was chosen instead of Haar-random unitaries.
   We thank the referee for raising this point. We only compute up to third moment of the density matrix. Therefore we only need a three design to make an unbiased estimation.

9. If the variational optimization was performed classically using pre-computed parameters, please state this explicitly.
   We thank the referee for raising this point and have further stressed this in the caption of Fig. 5.

10. The manuscript states that the decoding circuit was optimized locally on a classical computer. Does this imply that all experimental inputs were included in the optimization? If so, please mention this either in the main text or in the Supplementary Material.
    We thank the referee for raising this point and added a few sections in the appendix regarding the classical optimization method to make it even more scalable.

11. It is still unclear whether the dephasing model used was local or global. A brief clarification would be helpful.
    Dephasing is a tensor product of local dephasing on each site. We have now emphasized it in the introduction as well as caption of Fig. 1.

12. In the Supplementary Material, the stated 9% infidelity for correlators is not fully explained. Based on the provided error rates, I do not immediately see how this value is obtained. A brief explanation would suffice.
    We have explained this in the SM in the new version.

13. The caption of figure 13 appears to be missing an "L". I believe the authors meant $L = 4, 8$.
    Typo fixed!

Reviewer #3 (Remarks to the Author): I appreciate the effort the authors put into revising their manuscript in response to the referees' comments. Now the motivation of the proposed approach is clearer, and, in particular, the quality of the experimental data has improved significantly.

All these modifications improved the work overall, and I am now open to supporting the revised version for publication, provided that the authors address the points below.

1. It is still not clear whether the use of variational decoding for distinguishing mixed-state phases can be extended more broadly to classes of mixed-state phases beyond critical ground states under decoherence, the specific case tackled in this paper. This limits the relevance of the proposed method as a general diagnostic tool and makes its broader impact on the study of mixed-state phases somewhat uncertain. A more in-depth discussion in the outlook about possible generalizations would further strengthen the paper.

   We expect the variational decoding approach can be used to detect mixed-state phases that go significantly beyond the critical-state examples studied in this work. Many mixed-state phases admit a natural interpretation in terms of quantum error correction [1], such as mixed-state topological order and mixed-state symmetry protected topological (SPT) phases. The mixed-state phases are reflected in the robustness of encoded logical information under noise and recovery. Furthermore, the decoder is expected to be quasi-local within the same phase characterized by finite Markov length [2], which further supports the finite-depth variational decoding scheme we propose.

   As a concrete example, we illustrate how variational decoding is useful to detect different phases obtained through the one-dimensional SPT under decoherence. An SPT with open boundaries can be naturally interpreted as a QEC code with the gapless boundary modes. In this setting, our new numerical results (see SM) found that for strongly symmetric dephasing noise (where the SPT phase persists), the logical information remains highly recoverable, with an exact local decoder at the solvable point and a variational refinement that continues to work in the presence of a transverse field, whereas the decoding fidelity is lost upon moving toward the trivial phase.

   This suggests that the variational decoding scheme serves as a useful diagnostic of mixed-state SPT beyond analytically tractable limits. The approach extends naturally to higher-dimensional settings, where topological and fractonic phases already have a QEC interpretation, and where mixed-state phase transitions can be understood as transitions in recoverability. Therefore, variational decoding provides a general route to probing mixed-state phases where exact decoder is not known a priori, which includes the specific case of critical ground states under decoherence.

2. At the beginning of the last paragraph on page 3, the authors wrote, "In terms of the amplitude of $C^{(2)}_{XX}(l)$, we observe a significant increase under X dephasing ...". However, based on both Fig. 2 and Eq. (2), should we not expect it to saturate or to slightly decrease due to finite-size effects, as the authors point out in the caption? Maybe the authors mean X increases and Z decreases with respect to the identity channel? A clarification is needed.

   We thank the referee for their comment. We have now added "w.r.t. the identity channel"

3. Regarding the description of the behavior of F_e with respect to $pL^{1/2}$ for Z dephasing, the term "non-monotonic" may not be precise, as it appears to vary rather smoothly. I think the authors should clarify their meaning.

   Non-montotonic means that F_e decreases with L at small fixed $pL^{1/2}$ and increases with L at large fixed $pL^{1/2}$. We have reflected the referee's suggestion in the main text.

References

[1] Sang, Zou, Hsieh, "Mixed-state phases: renormalization and quantum error correction", *PRX* **14**, 031044 (2024).

[2] Sang, Hsieh, "Stability of Mixed-State Quantum Phases via Finite Markov Length", *PRL* **134**, 070403 (2025).

Reviewer #4 (Remarks to the Author): I co-reviewed this manuscript with one of the reviewers who provided the listed reports. This is part of the Nature Communications initiative to facilitate training in peer review and to provide appropriate recognition for Early Career Researchers who co-review manuscripts.

We thank the referee for their time.

Point-by-point response to the reviewers' comments

Reviewer #2 (Remarks to the Author):

> *All previous queries have been adequately addressed. I have no further comments and therefore recommend the manuscript for publication in Nature Communications.*

Response. We thank Reviewer #2 for their positive recommendation. No further changes were required.

Reviewer #3 (Remarks to the Author):

> *We have carefully examined the revised manuscript, the supplementary material, and the authors' responses to all reviewers' comments. Considering the authors' other responses and the additional material included in the revision, such as the application of variational decoding to symmetry-protected topological phases, we recommend publication in its present form.*
>
> *Regarding the scalability issue raised by Reviewer 1 regarding the variational approach, we find the authors' response convincing. That said, readers would benefit from an explicit clarification in the main text that the approach applies only to a local decoder and to correlations that evolve smoothly with the system size.*
>
> *As a final minor point, we would appreciate knowing the fitted slope for $C^{(2)}_{xx}(l)$ for the identity channel as well, since this would provide a better benchmark of processor performance. In particular, the exact numerical data appear to track the experimental data in both dephasing cases, but not in the identity-channel case in Figure 2(b).*

Response. We thank Reviewer #3 for their positive recommendation and for the two minor remaining suggestions, both of which we have implemented.

1. *Locality clarification in the main text.* We agree with the referee and have added an explicit clarification in the paragraph immediately preceding Fig. 5 of the main text:

   > *"We emphasize that this transfer-learning approach relies on the decoder being local and on the optimal-decoder parameters evolving smoothly with system size L, so that an optimum at $L-1$ provides a near-optimal seed at L."*

   This makes the assumption explicit in the main text and complements the more detailed discussion of the algorithm already given in the Methods section.

2. *Identity-channel slope.* We have refit the experimental $C^{(2)}_{XX}(l)$ data for the identity channel using the same protocol as for the X and Z dephasing cases (linear fit on the log scale with $l = 1, 2, 3$ at $L = 6$). The fitted slope is 0.21(8), compared to the field-theoretic prediction of 0.25. We have added this number to the body text and to the caption of Fig. 2(b). The deviation between the experimental fit and the exact-numerics curve in the identity-channel case is consistent with the level of device noise (logical-gate and SPAM error) discussed in the Supplementary Material; the fitted slope thus provides the benchmark of processor performance the referee asked for.

Reviewer #4 (Remarks to the Author):

I co-reviewed this manuscript with one of the reviewers who provided the listed reports. This is part of the Nature Communications initiative to facilitate training in peer review and to provide appropriate recognition for Early Career Researchers who co-review manuscripts.

Response. We thank Reviewer #4 for co-reviewing this manuscript as part of the Nature Communications early-career-researcher initiative.
